# Effects of Prebiotics and Probiotics on Symptoms of Depression and Anxiety in Clinically Diagnosed Samples: Systematic Review and Meta-analysis of Randomized Controlled Trials

**DOI:** 10.1093/nutrit/nuae177

**Published:** 2024-12-28

**Authors:** Afrida Asad, Megan Kirk, Sufen Zhu, Xue Dong, Min Gao

**Affiliations:** Nuffield Department of Primary Care Health Sciences, University of Oxford, Oxford OX2 6GG, United Kingdom; Nuffield Department of Population Health, University of Oxford, Oxford OX3 7LF, United Kingdom; Nuffield Department of Primary Care Health Sciences, University of Oxford, Oxford OX2 6GG, United Kingdom; NIHR Oxford Health Biomedical Research Centre, Warneford Hospital, Oxford OX3 7JX, United Kingdom; Nuffield Department of Primary Care Health Sciences, University of Oxford, Oxford OX2 6GG, United Kingdom; Nuffield Department of Primary Care Health Sciences, University of Oxford, Oxford OX2 6GG, United Kingdom; Nuffield Department of Primary Care Health Sciences, University of Oxford, Oxford OX2 6GG, United Kingdom; NIHR Oxford Health Biomedical Research Centre, Warneford Hospital, Oxford OX3 7JX, United Kingdom

**Keywords:** prebiotics, probiotics, depression, anxiety, meta-analysis

## Abstract

**Context:**

The use of prebiotics and probiotics as a treatment for psychiatric conditions has gained interest due to their potential to modulate the gut–brain axis. This review aims to assess the effectiveness of these interventions in reducing symptoms of depression and anxiety in psychiatric populations.

**Objective:**

The aim was to comprehensively review and appraise the effectiveness of prebiotic, probiotic, and synbiotic interventions in reducing clinical depression and anxiety symptoms.

**Data Sources:**

Systematic searches were conducted across Embase, Medline, PsycINFO, CINAHL, Cochrane Library, and Science Citation Index from database inception to May 22, 2023.

**Data Extraction:**

Randomized controlled trials investigating prebiotic, probiotic, or synbiotic interventions for treating clinical depression or anxiety symptoms in clinical samples were included. Data were extracted on study characteristics, intervention details, and outcome measures. The Cochrane Collaboration Tool was used to assess the risk of bias.

**Data Analysis:**

The standardized mean difference (SMD) was calculated using Hedge’s *g* as the metric of effect size. A random-effects model was applied to estimate pooled effect sizes with 95% CIs. Subgroup analyses were performed based on study characteristics, methodological factors, and intervention types. Sensitivity analyses excluded studies with a high risk of bias.

**Results:**

Twenty-three RCTs involving 1401 patients met the inclusion criteria, with 20 trials providing sufficient data for meta-analysis. Of these, 18 trials investigated probiotics for depression, 9 trials assessed probiotics for anxiety, and 3 trials examined prebiotics for depression. Probiotics demonstrated a significant reduction in depression symptoms (SMD: –0.96; 95% CI: –1.31, –0.61) and a moderate reduction in anxiety symptoms (SMD: –0.59; 95% CI: –0.98, –0.19). Prebiotics did not show a significant effect on depression (SMD: –0.28; 95% CI: –0.61, 0.04). High heterogeneity was observed across studies, and subgroup analyses indicated that study duration and probiotic formulations contributed to the variation in effect sizes.

**Conclusion:**

Probiotics showed substantial reductions in depression symptoms and moderate reductions in anxiety symptoms. Prebiotics showed a nonsignificant trend toward reducing depression. An adjunctive mental health treatment approach that diagnoses, monitors, and treats the gut microbiome alongside traditional pharmacological treatment holds promise for clinical practice.

**Systematic Review Registration:**

PROSPERO registration no. CRD42023424136.

## INTRODUCTION

Prior to the COVID-19 pandemic, an estimated 280 million people across the world had depression, affecting approximately 5% of adults worldwide.^1^ Anxiety disorders affect approximately 301 million individuals worldwide, with a prevalence of 4.05% in adults.^2^ Depression and anxiety have the highest rate of co-occurrence among all mental health conditions, with approximately 45%–67% of adults with clinical depression meeting criteria for at least 1 anxiety disorder, presenting challenges to treatment effectiveness.^3^ New data published in *The Lancet* have revealed a rapid rise in the global prevalence of depression and anxiety by over 27.6% and 25.6%, respectively (53.2 million additional cases for depression, 76.2 million additional cases for anxiety), due to the COVID-19 pandemic,^4^ contributing to higher health service use and a more complex clinical course. Antidepressants and psychological therapy are often the most commonly prescribed and effective treatments for mental disorders, but one-third to one-half of patients do not respond to these treatments, and the relapse rate following completion of treatment remains high.^5^ Access to treatment is restricted by growing waitlist times, misdiagnosed or underdiagnosed co-occurrence of depression and anxiety, shortage of qualified therapists,^4^ a lack of therapeutic alliance,^6^ financial costs associated with receiving treatment (eg, treatment-resistant depression),^7^ adherence factors (eg, low motivation, comorbid chronic illness), and perceived stigma of disclosure or medication use. Therefore, new approaches for managing symptoms of depression and anxiety with a great clinical effect are needed more urgently than ever before.

Emerging evidence suggests that gut microbiome disturbances are implicated in depression, and possibly with anxiety. The gut microbiota refers to the diverse array of microorganisms, including bacteria and fungi, residing in the human gastrointestinal system.^8^ In people with depression, Firmicutes, Actinobacteria, and Bacteroidetes are the most affected phyla,^9^ especially an increase in the Bacteroidetes-to-Firmicutes ratio, characterized by an enrichment of the genus *Bacteroides* and a depletion of the genera *Blautia*, *Faecalibacterium*, and *Coprococcus*.^10^^,^^11^ An increase in *Eggerthella* and decrease in *Sutterella* were also consistently demonstrated in people with depression and anxiety.^12^ Strong evidence highlights the interconnected, bidirectional mechanisms through which the gut microbiome interacts with the central nervous system of the brain, which is explained as the gut–brain axis (GBA) mechanism. Recent studies suggest that gut microbiota may alter the function of neurotransmitters and even produce neurotransmitters like serotonin and dopamine, which may directly affect depressive symptoms.^13^ On the other hand, abnormal enteric nervous system activity arising from intestinal pathology aggravates depression-related pathological changes by altering gut secretion, immune defenses, motility, and permeability.^14^ The microbiome also modulates the stress response through the vagus nerve, and produces neuroprotective factors, which collectively decrease depressive and anxiety symptoms.^15^

Given the evidence implicating the GBA in depression and anxiety, there has been interest in developing treatments that target the GBA. Probiotics are living microorganisms that, when consumed, have a beneficial impact on host health, and prebiotics are nondigestible food components that provide health benefits to the host by influencing microbiota modulation.^16^ Synbiotics are dietary components containing both pre- and probiotics. The impact of probiotics on metabolism can also be promoted by prebiotics, which stimulates the proper growth of probiotic bacteria and can support the gut–brain interaction.

Over the last decade, the proliferation of systematic reviews and meta-analyses on the use of prebiotics, probiotics, and their combination for alleviating mental health symptoms can be attributed to an expanded understanding of the role of the GBA in mental illness, the complexity of treating psychiatric disorders, and the need for alternative treatments.^17^ Despite the rapid research interest in this field, the overall effects of gut microbiota treatments for depression and anxiety remain inconclusive and difficult to interpret. This is largely due to outdated or limited searches,^18^^,^^19^ high heterogeneity in population inclusion criteria (clinically depressed population^19^^,^^20^ vs healthy population^21^^,^^22^ vs comorbid population^23^), variation in intervention type (adjunctive/multicomponent vs stand-alone treatment), lack of evaluation or inclusion of the effects on anxiety, no clear information on what intervention factors influence the outcomes, and issues with reporting quality (eg, no publication bias, meta-analysis on 3 randomized controlled trials [RCTs]).^19^^,^^24^^,^^25^

A 2019 review of 34 trials examining prebiotics and probiotics for depression and anxiety found that sample type was a significant moderator (*P* < .01)^26^; larger intervention effects were observed in clinical/medical samples (*d *= –0.45, *P* < .001) compared with community populations (*d *= –0.09, *P *= .09), where lower levels of depression symptom severity are expected.^26^ In the exploratory analysis of 4 trials with clinically diagnosed major depression, there was an increased treatment effect (*d *= 0.73, *P* < .001)^26^ found for probiotics and depression compared with nonclinical samples. However, clinical mental health samples only represented 14.7% (*n *= 4) of the 34 trials included in the review, limiting the interpretability of gut microbiome interventions in a clinical context. There remains a large gap in understanding the clinical relevance of gut microbiota interventions to treat depression and anxiety symptoms.

The most current evidence comes from a recent 2023 meta-analysis of 13 RCTs with 786 participants estimating the effectiveness of pre-, pro-, and synbiotics use on clinical depression symptoms, which found an overall small effect-size reduction in depression severity (standardized mean difference [SMD] = –0.34; 95% CI: –0.45, –0.22) in favor of the treatment group compared with placebo controls.^20^ Subgroup analysis revealed that only probiotics, both single and multiple strain, were associated with significant, small effect-size reductions in depression severity (*P *< .05) compared with placebo. In addition, meta-regression analysis revealed a larger reduction in depression symptoms (coefficient = 1.925, *P *= .026) in studies that had fewer females (<70% females). Treatment duration, intervention agent, intervention type, and evaluation time point did not emerge as effect moderators. Overall, this 2023 review provides the most up-to-date evidence for clinically depressed populations regarding the effects of prebiotics, probiotics, and synbiotics for depression.

To the authors' knowledge, this meta-analytic review is the first to examine the effectiveness of prebiotic, probiotic, and synbiotic treatments, with or without pharmacotherapy, in reducing depression and anxiety symptoms exclusively among clinically diagnosed samples. The review expands upon the work by Liu et al^26^ by including 17 additional trials with clinically diagnosed samples. It also builds on the review by Zhang et al^20^ by incorporating analysis of 10 more trials and conducting comprehensive subgroup analyses to gain new insights into treatment effects and identify potential sources of heterogeneity of prebiotics, probiotics, or synbiotics for clinical depression and anxiety.

The primary objectives of this systematic review and meta-analysis were 4-fold: (1) to synthesize and evaluate the extant evidence from RCTs investigating prebiotics, probiotics, and synbiotics in alleviating symptoms of depression and anxiety among clinically diagnosed populations with depression and/or anxiety; (2) to summarize meta-analytic findings of the main effects; (3) to comprehensively estimate what intervention factors may influence treatment effects; and (4) to establish future recommendations for the use of gut microbiota treatment for mental health disorders.

## METHODS

This systematic review was conducted in accordance with the Preferred Reporting Items for Systematic Reviews and Meta-Analyses (PRISMA) statement. The protocol for the systematic review was prospectively registered with the International Prospective Register of Systematic Reviews (PROSPERO, CRD42023424136).

### Search Strategy and Eligibility Criteria

The search strategy was developed following the key concepts of prebiotic, probiotics, synbiotic, depression, and anxiety. Searches were conducted on Embase (Ovid; 1974–present), Medline (OvidSP; 1946–present), PsycINFO (OvidSP; 1806–present), CINAHL (EBSCOhost*;* 1982–present), the Cochrane Database of Systematic Reviews and Cochrane Central Register of Controlled Trials (Cochrane Library, Wiley; Issue 5 of 12, May 2023), and Science Citation Index (Web of Science Core Collection; 1900–present) from inception to May 22, 2023, using search terms that included synonyms of “pre/probiotic” AND “depression” or “anxiety”. A combination of free-text (title/abstract/author) key words and MeSH (Medical Subject Heading) terms was developed to describe the key concepts (Table S1). Studies were restricted to the English language only.

The eligibility criteria for the studies included in this meta-analysis are summarized in Table 1. Cluster and individual RCTs that involved adults diagnosed with depression and/or anxiety were assessed, where prebiotics and/or probiotics were the only active components administered in the intervention group. A validated search filter was applied to restrict RCTs; the references were exported to Covidence for screening.

**Table 1.** PICOS Criteria for Inclusion of Studies

Parameter	Criteria
Participants (P)	Adults aged 18–65 years clinically diagnosed with depression and/or anxiety by a clinician (eg, ICD-10 or DSM-V criteria) or meeting threshold criteria (eg, MADRS ≥13).
Interventions (I)	Prebiotics and/or probiotics as the only active components in the intervention group.
Comparisons (C)	Placebo and/or treatment as usual (eg, antidepressants), with the same active components administered in both treatment and placebo groups, except for the addition of prebiotics and probiotics in the intervention group.
Outcomes (O)	Changes in depression and/or anxiety scores from baseline to postintervention.
Study design (S)	Cluster- and individual RCTs of any duration. A validated search filter was used to restrict to RCTs, and references were screened using Covidence.

Abbreviations: ICD-10, International Classification of Diseases, Tenth Revision; DSM-V, Diagnostic and Statistical Manual of Mental Disorders, Fifth Edition; MADRS, Montgomery–Åsberg Depression Rating Scale; RCT, randomized controlled trial.

### Screening and Data Extraction

**Figure 1.**
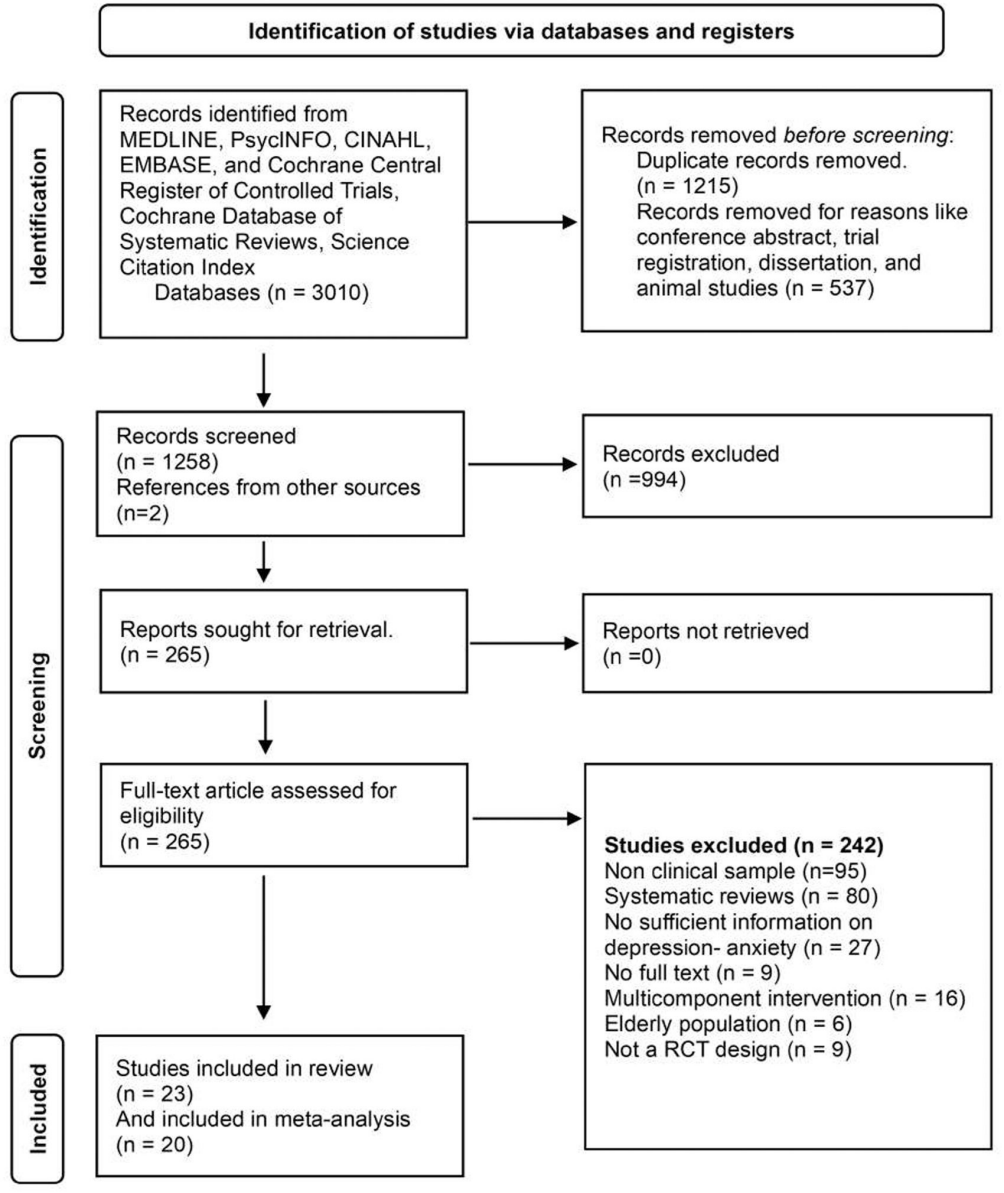
PRISMA (Preferred Reporting Items for Systematic Reviews and Meta-Analyses) Flowchart of Included Trials and Reasons for Exclusion. Abbreviation: RCT, randomized controlled trial

One lead reviewer (A.A.) and a second reviewer (M.G./M.K.) independently screened potentially eligible studies based on title and abstract, followed by full-text screening where applicable. Dual screening was conducted in 100% of the publications using Covidence systematic review software, Veritas Health Innovation, Melbourne, Australia. Available at www.covidence.org. Disagreements were resolved in consultation with third reviewer until consensus was achieved. The study selection process was recorded in Covidence and all decisions for inclusion and exclusion were recorded. Cross-referencing of bibliographies of trials selected for inclusion and manual searches online were conducted. Where possible, the corresponding authors were contacted for missing data or raw values. The summary of the screening and selection process is shown in Figure 1.

An a priori data-extraction form was developed by the lead authors (A.A./M.K.) based on (1) previous systematic reviews of the topic and (2) the PICOS (Participants, Intervention, Comparison, Outcomes, Study design) framework^27^ and was pilot-tested with 3 full-text publications by 2 reviewers (A.A./S.Z.) independently for accuracy and completeness. The data-extraction form included information regarding authors, date, country of publication, study design, sample demographic characteristics (age, sex, comorbidities), diagnosis type (clinically diagnosed vs self-report), change in depression and anxiety score assessment (tools; self-assessed or clinician assessed), sample size (baseline, postintervention), compliance to intervention, effect of the intervention vs the control group, and details of intervention factors (type, composition, dose). The mean and SD of depression and/or anxiety score (separately mentioned) from baseline and postintervention were extracted from the full-text studies. Where studies included multiple measures for the same outcome, the first listed outcome was prioritized to maintain consistency across studies. The extracted data were independently reviewed manually by 2 reviewers (A.A./S.Z.) for all included studies and further cross-checked by senior authors (M.K./M.G.) for accuracy. No automated tools or approaches were used. Data were stored as an Excel database.

### Risk of Bias and Certainty of Evidence Assessments

The quality assessment of the selected studies for review was conducted following the Cochrane Collaboration Tool for assessing bias. The tool consists of 6 items and each item evaluation gave a subjective assessment of external and internal validity of the studies, labeling them as “low,” “some concern,” and “high” risk studies. Two researchers (A.A./S.Z.) independently assessed bias and any discrepancies or disagreements were resolved in consultation with the third author through discussion until consensus was reached. Certainty of evidence was evaluated using the Grading of Recommendation, Assessment and Evaluation (GRADE) approach. Two reviewers (A.A./M.K.) independently rated the certainty of evidence for each significant outcome. The quality of evidence was assessed using factors such as study design, risk of bias, inconsistency, indirectness, imprecision, and publication bias.

### Data Analysis

A narrative synthesis of outcomes across the studies was undertaken and summary tables were used to describe the characteristics of the populations, interventions, comparison groups, and outcomes of studies included in the review. Studies were divided into groups according to the type of intervention, classified as prebiotics and probiotics.

The main effect size was the SMD, calculated using Hedge's *g*. Negative SMD values reflected reduced depression and anxiety levels in favor of the treatment group compared with a comparator. Forest plots were used to illustrate results graphically. The random-effects model pooled the results, assuming the true effect sizes across different studies follow a random distribution, accounting for both within-study and between-study variability. For the random-effects meta-analysis model, the restricted maximum likelihood (REML) method was used for estimating between-study variance. Confidence intervals for the summary effect were calculated using the Wald-type method. Results are presented as SMDs (Hedges' *g*) with 95% CIs. SMD values of ±0.20, ±0.50, and ±0.80 represented small, moderate, and large effects, respectively.^28^ Studies that did not assess depression and anxiety separately were excluded in the meta-analysis. Heterogeneity was assessed using the *I*^2^ statistic, with low, moderate, and high heterogeneity indicated at 25%, 50%, and 75%, respectively.^29^

Exploratory subgroup analyses were performed ad hoc based on various study characteristics, including intervention types (adjunctive with antidepressants vs stand-alone prebiotic/probiotic), clinically diagnosed cases (International Classification of Diseases/Diagnostic and Statistical Manual of Mental Disorders [ICD/DSM] criteria vs self-reported), change in score assessment method (self vs clinical), strain type (single strain vs multi-strain), intervention duration (<8 weeks vs ≥8 weeks), and presence of comorbidities (with vs without). Sensitivity analyses were conducted to repeat the main analysis after excluding studies at high risk of bias. Given the uncertainty surrounding the body of literature before conducting the analysis, subgroup and sensitivity analyses were not prespecified. Trim-and-fill analysis and Egger’s regression were performed to assess for publication bias using STATA/SE version 18 software package (StataCorp LLC, College Station, TX, USA).

### Patient and Public Involvement

In collaboration with the McPin Foundation, a 12-member panel with lived experience of mental illness and comorbid chronic disease conditions met quarterly to identify the priorities and research agenda of the Oxford Health Biomedical Research Centre (BRC) Preventing Multiple Morbidities theme. The Patient and Public Involvement panel provided suggestions on a public-facing lay summary and infographic to be circulated to community partners and stakeholders of this manuscript.

## RESULTS

A total of 1258 publications were identified through the initial database search (Figure 1). Two additional publications were identified through manual search of references. Following deduplication and title/abstract screening, 994 publications were removed based on prespecified criteria. Overall, 265 studies were retrieved for full-text screening. Based on prespecified inclusion criteria, 242 studies were excluded, with reasons recorded (Figure 1). A total of 23 publications met the inclusion criteria and are summarized in the review (Table 2). Of these, 20 studies provided sufficient data to be included in the meta-analysis.^30–49^ Among the 3 excluded studies, 1 presented ordinal logistic regression analysis with no raw mean (SD) score,^50^ and 2 did not provide the raw pre-post or between-group change scores (mean, SD) to be included in the meta-analysis performed.^51^^,^^52^

**Table 2.** Study Characteristics of Included Articles (*n* = 23)

First author, year	Country	No.	Study population	Age (mean), % female	Prebiotic compound(s)/probiotic microbe(s), dosage (form, frequency, timing)	Duration	Clinical outcome(s)	Clinical measure	Compliance	Control/blinding
Prebiotics (*n* = 3)										
Heidarzadeh-Rad,^a^ 2020^30^	Iran	52	Mild to moderate melancholic depression for at least 1 month, and taking antidepressant medications	36.47 y, 70%	80% galacto-oligosaccharide (GOS) per sachet/d	8 wk	Dep	BDI	NR	TAU/double-blind
Kazemi,^a^ 2019^31^	Iran	72	Mild to moderate major depressed patients, and taking antidepressant drugs for 3 months or more prior	36.7 y, 70.8%	GOS	8 wk	Dep	BDI (self-report)	NR	TAU/double-blind
Tarutani,^a^ 2022^32^	Japan	20	MDD (ICD-10)	53.6 y, 85%	4G-beta-D-galactosylsucrose (LS) syrup; 7 g syrup/d (contains 3.2 g LS)	24 wk	Dep	QIDS	NR	TAU, double-blind
Probiotics (*n* = 23)										
Akkasheh,^a^ 2016^33^	Iran	40	MDD (DSM-IV)	37.3 y, 85%	*Lactobacillus acidophilus* L. casei and *Bifidobacterium bifidum* (2 × 10^9^ CFU/g); 1 capsule/d + citalopram	8 wk	Dep	BDI	90%	TAU, double-blind
Baião,^a^ 2022^34^	UK	71	Clinical psychologist diagnosed mild to moderate depression (PHQ-9 score 5-19)	28.8 y, 63%	14 MS^b^ (2 × 10^9^ CFU/g); 4 capsules/d in the morning	4 wk	Dep, Anx	PHQ-9, STAI-T	NR	Placebo, double-blind
Browne, 2021^51^	Netherlands	40	Mild depressive symptoms and/or anxiety in second/third trimester of uncomplicated pregnancy	30.6 y, 100%	Ecological barrier,^c^ (2.5 × 10^9^ CFU/g); 2-g sachet/d	8 wk	Dep, Anx	EPDS, STAI-S	98%	Placebo, double-blind
Chahwan,^a^ 2019^35^	Australia	71	Depressive symptoms (BDI-II score >12) by the research team	36 y, 69%	2 sachets/d of 2 g of freeze-dried probiotic powder mixture of ecological barrier^c^ (2.5 × 10^9^ CFU/g)	8 wk	Dep, Anx	BDI, DASS-Anxiety	NR	Placebo, triple blinded
Eskandarzadeh,^a^ 2021^36^	Iran	36	Diagnosis of GAD based on DSM-V, SCID-I interviews by psychiatrist	33.9 y, 81%	*B longum*, *B bifidum*, *B lactis*, and *L acidophilus* (18 × 10^9^ CFU/g); 1 capsule/d during lunch + sertraline (25 mg/d)	8 wk	Anx	HAM-A	NR	TAU, double-blind
Ghorbani,^a^ 2018^37^	Iran	40	Moderate depression (DSM-V) by expert psychologist interview	35 y, 70%	500 mg probiotic (*L casaei* 3 × 10^8^ CFU/g*, L acidophilus* 2 × 10^8^ CFU/g, *L bulgaricus* 2 × 10^9^ CFU/g, *L rhamnosus* 3 × 10^8^ CFU/g, *B breve* 2 × 10^8^ CFU/g, *B longum* 1 × 10^9^ CFU/g, *S thermophilus* 3 × 10^8^ CFU/g) + 100 mg prebiotic (fructo-oligosaccharide), 1 capsule daily + fluoxetine (20 mg/d) for 4 wk	8 wk	Dep	HAM-D	NR	TAU, double-blind
Heidarzadeh-Rad, 2020^30^	Iran	53	Mild to moderate melancholic depression for at least 1 month, and taking antidepressant medications	36.6 y, 70%	*L helveticus* and *B longum* (≥2 × 10^9^ CFU/g), 1 sachet 5 mg/d	8 wk	Dep	BDI	91.90%	TAU, double-blind
Kazemi,^a^ 2019^31^	Iran	74	Mild to moderate major depressed patients, and taking antidepressant drugs for 3 months or more prior	36 y, 71%	*L helveticus + B longum* (≥2 × 10^9^ CFU/g) 5 mg/d	8 wk	Dep	BDI (self-report)	91.90%	TAU, double-blind
Kotelnicka,^a^ 2023^38^	Poland	60	Clinically diagnosed depression (MADRS ≥ 13)	34.93 y, 85.4%	Probiotic mixture (*L helveticus* and *B longum*) (3 × 10^9^ CFU/g) + amitriptyline	8 wk	Dep, Anx	MADRS, DASS- Anxiety	100.00%	TAU, double-blind
Majeed,^a^ 2018^39^	India	40	MDD (DSM-IV) with mild to moderate IBS in severity with possible sleep, pain and dementia-associated comorbidities (Rome III Diagnostic Criteria for IBS)	42.08 y, 85%	*Bacillus coagulans* (2 × 10^9^ CFU/d), 1 tablet (600 mg)/d	12 wk (90 d)	Dep	HAM-D	60%	Placebo, double-blind
Miyaoka,^a^ 2018^40^	Japan	40	TRD, DSM-IV-TR	43 y, 52%	*Clostridium butyricum* MIYAIRI 588 (CBM588), 60 mg/d	8 wk	Dep, Anx	HAM-D, BAI	NR	TAU (SSRI or SNRI), open label
Nikolova,^a^ 2023^41^	UK	49	MDD (HAMD-17)	31.7 y, 80%	14 MS^b^(2 × 10^9^ CFU/capsule), 4 capsules/d	8 wk	Dep	HAM-D, HAM-A	97.20%	TAU, double-blind
Pinto-Sanchez,^a^ 2017^42^	Canada	44	Mild to moderate anxiety and/or depression (HAD-anxiety or HAD-depression [HAD-D] with a diagnosis of IBS with diarrhea or mixed-stool pattern (Rome III criteria)	43.25 y, 54%	*Bifidobacterium longum* (1.0 × 10^10^ CFU/g) in sachet; 1 sachet/d to mix with lactose-free milk/rice milk/soy milk	6 wk	Dep, Anx	HAD-D, HAD-A, HAM-D	60.00%	Placebo, double-blind
Romijn,^a^ 2017^43^	New Zealand	79	Depressive symptoms, QIDS-SR	35.5 y, 78%	*L helveticus* and *B longum* (>3 × 10^9^ CFU) per 1.5-g sachet, 1 sachet/d + psychological therapy	8 wk	Dep, Anx	MADRS, DASS	97%	Placebo, double-blind (psychological therapy)
Rudzki,^a^ 2019^44^	Poland	60	MDD (DSM-IV-TR)	39 y, 71%	*L plantarum* (10 × 10^9^ CFU/capsule), 2 capsules/d	8 wk	Dep	HAM-D	NR	TAU (SSRI), double-blind
Schaub, 2022^52^	Switzerland	60	Depressive episodes (ICD-10)	39 y, 54%	8 strains (*S thermophilus*, *B breve*, *B longum*, *B infantis*, *L acidophilus*, *L plantarum*, *L paracasei*, *L helveticus*) (9 × 10^9^ CFU/pill/d) to mix with noncarbonated drink	4 wk	Dep, Anx	HDRS, STAI-S	88%	TAU, double-blind
Tian,^a^ 2023^46^	China	28	Mild to moderate MDD (DSM-IV criteria)	43.5 y, 71.4%	*B breve*, *B longum*, and *P acidilactici* (4 × 10^9^ CFU/g), sachet/d	4 wk	Dep	HAM-D	NR	TAU, double-blind
Tian,^a^ 2022^45^	China	51	Patients with MDD (score >14 in HDRS-24)	49.55 y, 66%	*Bifidobacterium breve* (1 × 10^10^ CFU/g), 1 sachet/d	4 wk	Dep	HDRS-24	NR	TAU (SSRI), double-blind
Wei,^a^ 2022^47^	China	60	Clinically diagnosed with SIBO with depression and diabetes	43.45 y, 45%	Lactic acid bacteria capsule, dose of 0.66 g/time, 3 times/d, + escitalopram orally (10 mg/d)	1 wk	Dep, Anx	SDS, SAS	NR	TAU, triple blind
Zeng, 2022^50^	China	72	BPD with manic or major depressive episode (DSM-V) diagnosed by 2 senior psychiatrists	21.6 y, NR	Probiotic capsule (live combined *Bifidobacterium*, *Lactobacillus*, and *Enterococcus*) (1 × 10^7^ CFU/capsule), 6 capsules/d	12 wk	Dep, Anx	HAM-D, HAM-A	NR	TAU, double-blind
Zhang,^a^ 2021^48^	China	69	Psychiatrist-diagnosed depression (DSM-V) and constipation (Rome IV criteria)	47.5 y, 63%	*Lacticaseibacillus paracasei* (1.0 × 10^10^ CFU/100 mL); 1 bottle of fermented dairy beverage/d after lunch	9 wk	Dep	BDI	84%	Placebo, double-blind
Zhu,^a^ 2023^49^	China	60	Clinically diagnosed (HAMA-14, HDRS-17)	22.4 y, 50%	*L plantarum* (1.5 × 10^10^ CFU/g) sachet, 2 sachets/d	3 wk	Dep, Anx	HDRS-17, HAMA-14	NR	Placebo, double-blind

a Included in meta-analytic calculations.

b 14 MS: This refers to 14 multi-strains of bacteria, including *Bacillus subtilis, B. bifidum, B. breve, B. infantis, B. longum, L. acidophilus, L. delbrueckii* ssp*. bulgaricus, L. casei, L. plantarum, L. rhamnosus, L. helveticus, L. salivarius, Lactococcus lactis* ssp*. lactis*, and *Streptococcus thermophilus*.

c Ecological barrier: This refers to specific strains of bacteria, including *B. bifidum, B. lactis, Bifidobacterium lactis, L. acidophilus, L. brevis, L. casei, L. salivarius*, and *Lactococcus lactis*. In cases where data for multiple outcome measures were available in a given study, the primary effect measures were incorporated into the meta-analysis. The number, mean age, and percentage of female participants included in the relevant analyses, rather than for the entire study sample, were presented and incorporated into moderator analyses whenever such data were available and applicable.

Abbreviations: Anx, anxiety; BAI, Beck Anxiety Inventory; BDI, Beck Depression Inventory; BPD, borderline personality disorder; CFU, colony-forming units; DASS, Depression Anxiety and Stress Scale; Dep, depression; DSM, Diagnostic and Statistical Manual of Mental Disorders; EPDS, Edinburgh Postnatal Depression Scale; GAD, generalized anxiety disorder; GOS, galacto-oligosaccharide; HADS-A, Hospital Anxiety and Depression Scale–Anxiety subscale; HADS-D, Hospital Anxiety and Depression Scale–Depression subscale; HAM-A, Hamilton Rating Scale for Anxiety; HAMD, Hamilton Depression Rating Scale; HAMD-17, Hamilton Depression Rating Scale, 17-item; HDRS, Hamilton Depression Rating Scale; HDRS-17, Hamilton Depression Rating Scale, 17-item; IBS, irritable bowel syndrome; ICD, International Classification of Diseases; MADRS, Montgomery–Åsberg Depression Rating Scale; MDD, major depressive disorder; NR, not reported; PHQ-9, 9-item Patient Health Questionnaire (PHQ-9 incorporates DSM-IV depression diagnostic); POMS, Profile of Mood States; QIDS-SR, Quick Inventory of Depressive Symptomatology Self-Report; SAS, Self-rating Anxiety Scale; SDS, Sheehan Disability Scale; SIBO, small intestinal bacterial overgrowth; SNRI, serotonin-noradrenalin reuptake inhibitor; SSRI, selective serotonin reuptake inhibitor; STAI, State-Trait Anxiety Inventory; STAI-S, State-Trait Anxiety Inventory-State subscale; STAI-T, State-Trait Anxiety Inventory-Trait subscale; TAU, treatment as usual (usual antidepressants, eg, sertraline, fluoxetine, citalopram, or amitriptyline); TRD, treatment-resistant depression.

### Study Characteristics

Table 2 outlines characteristics of 23 included publications. Twenty-two studies examined probiotics,^30^^,^^31^^,^^33–52^ 3 studies (13.6%) investigated prebiotics,^30–32^ and 1 study investigated synbiotics.^37^ Overall, 21 studies were 2-arm RCTs (92%) with a placebo control group, while 2 studies^30^^,^^31^ conducted 3-arm RCTs with separate probiotic, prebiotic, and placebo-controlled interventions. A total of 1280 participants were included in all 23 studies with small trials (range: *n *= 20–79) and 144 and 1197 participants receiving a prebiotic or probiotic intervention, respectively. The mean age ranged from 21 to 53 years, and most studies (92%) included predominantly female samples (range: 54%–100%). Most studies were conducted in China (*n *= 6) and Europe (*n *= 6), followed by Iran (*n *= 4).

### Risk of Bias Assessment

**Figure 2.**
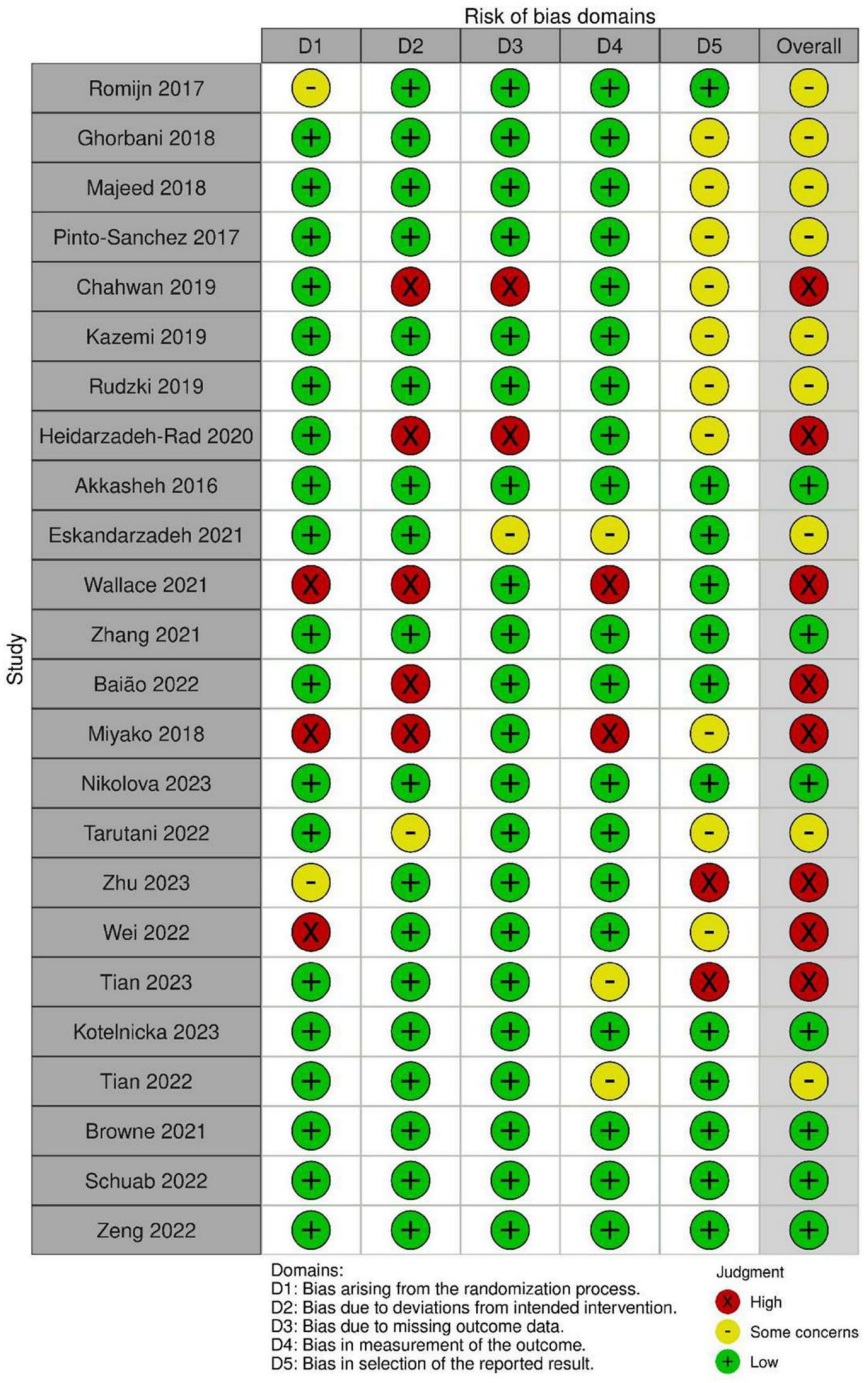
Traffic Light Plot Showing Individual Study’s Risk of Bias Assessment

Risk of bias was evaluated using the Cochrane Collaboration’s Risk of Bias 2 tool. Seven studies (30.4%) were judged to be at low risk of bias^33^^,^^38^^,^^41^^,^^48^^,^^50–52^ and 7 (30.4%) studies at high-risk bias,^30^^,^^35^^,^^34^^,^^40^^,^^46^^,^^47^^,^^49^ leaving 9 (39.1%) studies with overall some risk of bias concern.^31^^,^^32^^,^^36^^,^^37^^,^^39^^,^^42–45^ The summary risk of bias of all studies can be found in Figure S1 and individual risk of bias is shown in Figure 2.

### Meta-analysis

#### Prebiotics and Depression Severity

**Figure 3.**
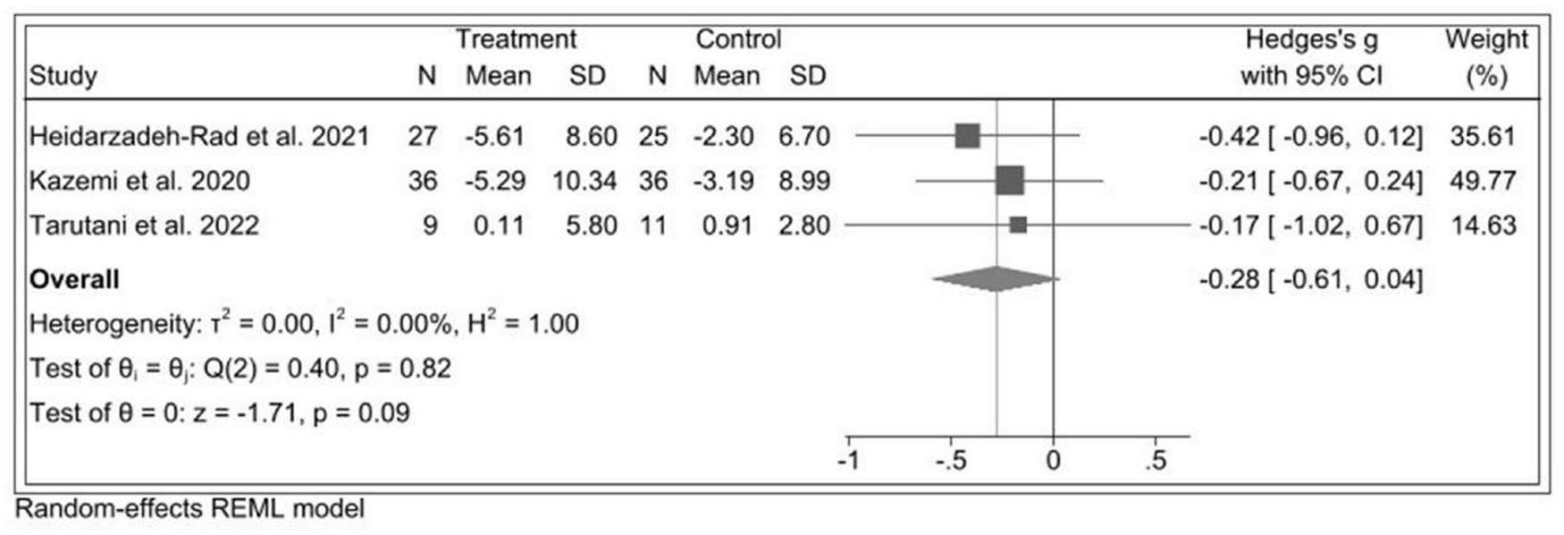
Forest Plot of Prebiotic Intervention to Reduce Depression Severity. Abbreviation: REML, restricted maximum likelihood

As shown in Figure 3, across 3 prebiotic RCTs of 144 participants,^30–32^ the estimated pooled effect of prebiotic in reducing depression was SMD* = –*0.28 (95% CI: –0.61, 0.04) and showed a nonsignificant difference (*P *= .09) between the prebiotic and control group (Figure 3), with no heterogeneity being observed (*I^2^* = 0%, *P *= .82).

In sensitivity analysis after excluding studies with a high risk of bias,^30^ the effect size decreased and remained nonsignificant (Figure S2). In analyses of publication bias, Egger's regression test indicated that there was no significant publication bias (intercept = 0.23, *P *= .93), and the adjusted effect size produced with the trim-and-fill method was similar in absolute value terms (adjusted SMD = −0.28; 95% CI: −0.605, −0.041) and the corresponding funnel plot of effect sizes was slightly asymmetrical (Figure S5).

#### Probiotics and Depression Severity

**Figure 4.**
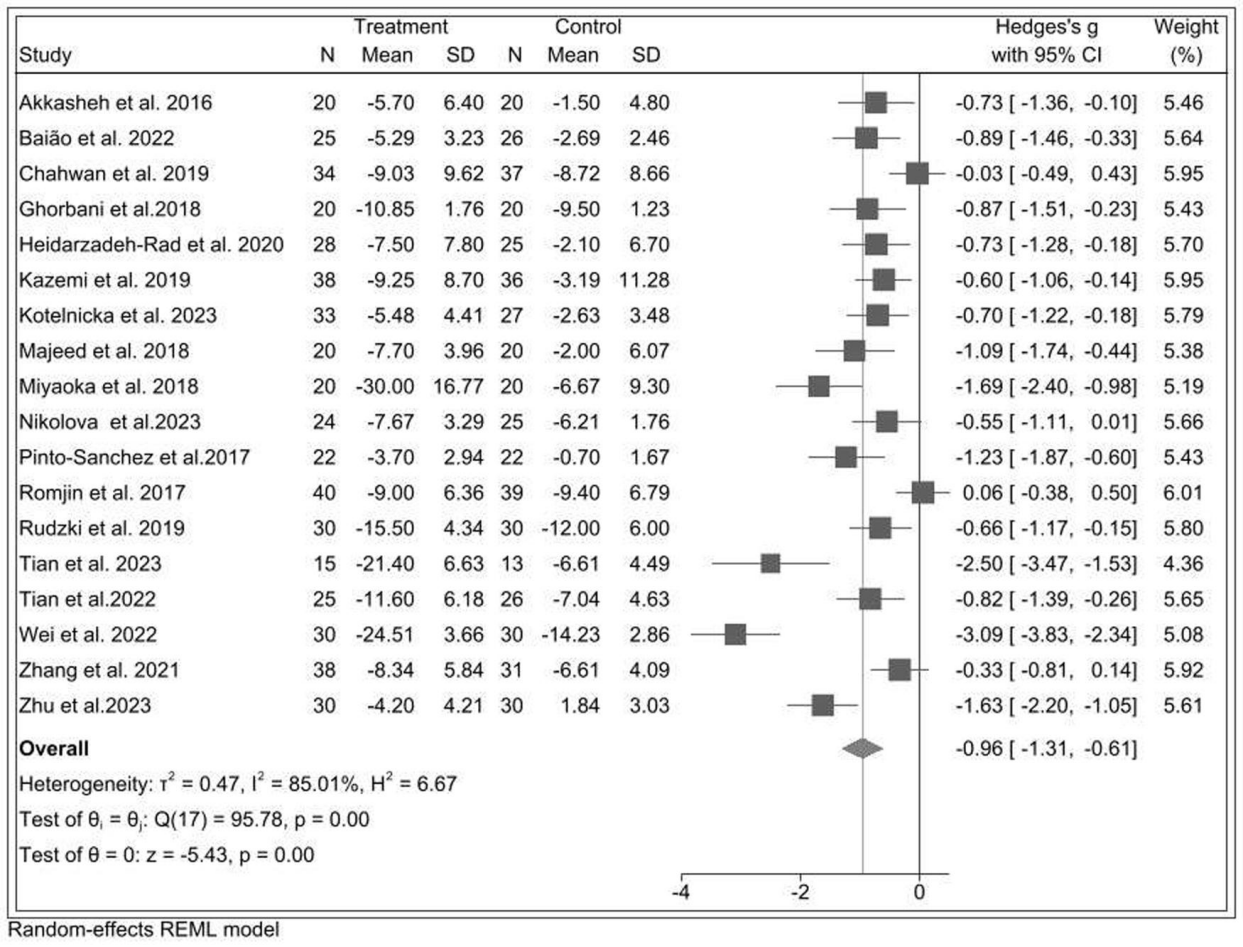
Forest Plot of Probiotic Interventions in Relation to Depressive Symptoms. Abbreviation: REML, restricted maximum likelihood

In Figure 4, across 18 probiotic RCTs of 969 participants,^30^^,^^31^^,^^33–35^^,^^37–49^ the pooled effect of probiotics in reducing depression score was SMD* = –*0.96 (95% CI: –1.31, –0.61) between the probiotic and control group, with high between-study heterogeneity (*I^2^* = 85%, *P *< .001), yielding a large effect-size reduction.

In sensitivity analysis after excluding 7 studies with a high risk of bias,^30^^,^^35^^,^^34^^,^^40^^,^^46^^,^^47^^,^^49^ the pooled effect size was reduced by nearly 32% (SMD: –0.64; 95% CI: –0.86, –0.42), with moderate heterogeneity (*I^2^* = 43.95%, *P *= .06) (Figure S3). Evidence of publication bias was observed with Egger’s regression test (intercept: –10.86, *P *< .001), and the pooled effect size with the trim-and-fill method adjusting for imputed missing effects did not change from the pooled effect size. The funnel plot was slightly asymmetrical (Figure S6).

#### Probiotics and Anxiety Severity

**Figure 5.**
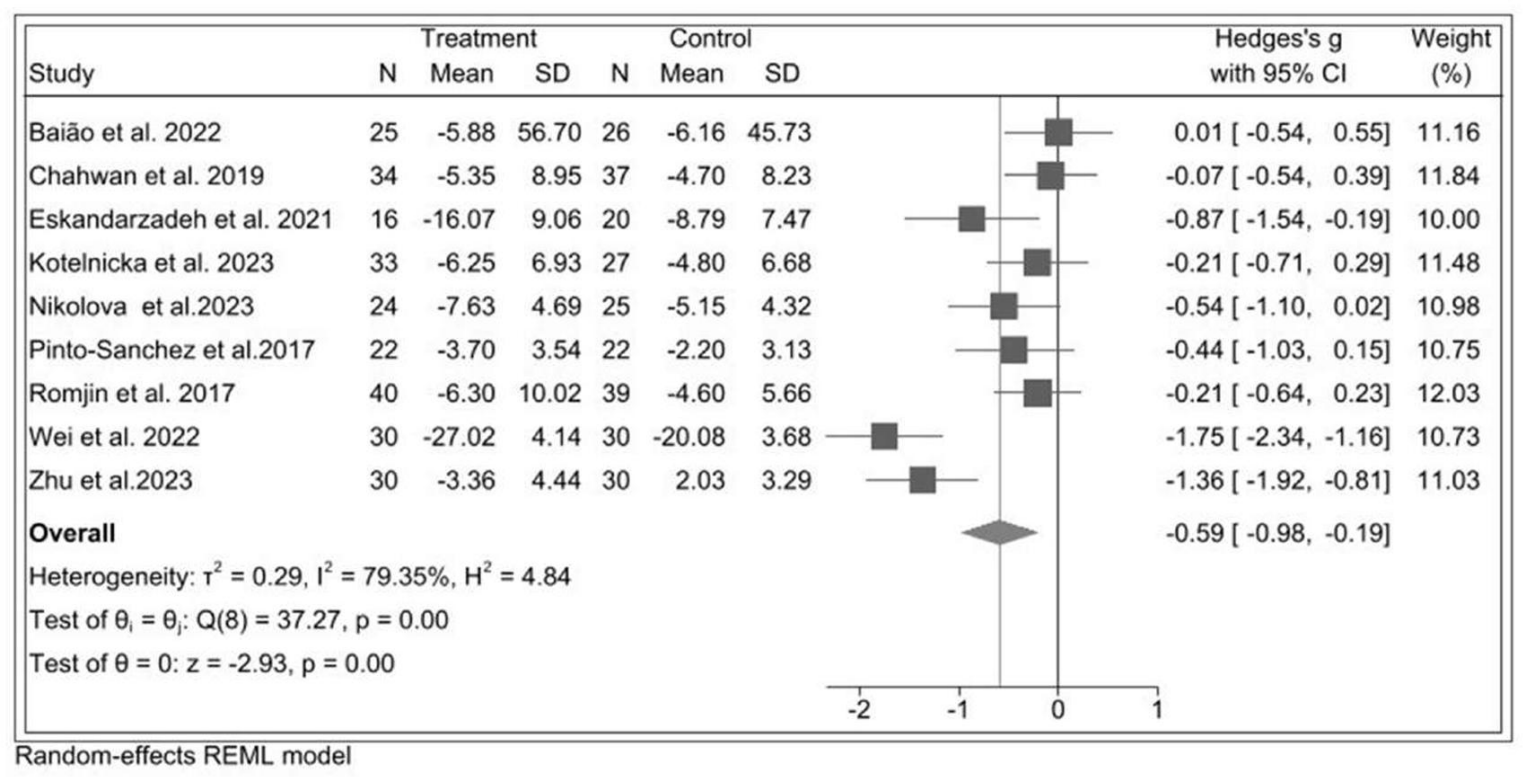
Forest Plot of Probiotic Intervention in Relation to Anxiety Symptoms. Abbreviation: REML, restricted maximum likelihood

Across 9 different probiotic RCTs including 510 participants,^34–36^^,^^38^^,^^41–43^^,^^47^^,^^49^ the estimated pooled effect of probiotic in reducing anxiety score was SMD* = –*0.59 (95% CI: –0.98, –0.19) between the probiotic and control group, with high heterogeneity (*I^2^* = 79.35%, *P* = .00) (Figure 5).

In sensitivity analysis after excluding 4 studies with a high risk of bias,^34^^,^^35^^,^^47^^,^^49^ the effect size was reduced by 20% in studies (SMD: –0.39; 95% CI: –0.63, –0.15), with no heterogeneity (*I^2^* = 0%, *P *= .49) (Figure S4). A significant publication bias was observed with Egger’s regression test (intercept: –9.62, *P *= .00), but the pooled effect size with the trim-and-fill method adjusting for imputed missing effects remained unchanged (adjusted SMD = −0.524; 95% CI: −0.701, −0.346). The funnel plot of effect sizes was asymmetrical (Figure S7).

### Subgroup Analysis

Subgroup analysis revealed varying effect sizes (SMD) for depression reduction with probiotics (Table 3; Figures S8–S13). Cochran's *Q* statistic was calculated to assess the degree of heterogeneity among effect sizes across the studies included in the meta-analysis. Studies with shorter duration (<8 weeks) saw larger effect size reductions in depression (SMD: –1.54; 95% CI: –2.18, –0.90) than those with longer interventions (SMD: –0.59; 95% CI: –0.86, –0.33), giving a significant *Q* value of 7.10 (*P* < .05). The pooled effect size was reduced by nearly 33% in studies after excluding 7 high-risk studies (SMD: –0.64; 95% CI: –0.86, –0.42) with moderate heterogeneity (*I^2^* = 43.95%, *P *= .06) (Figure S3). Evidence of substantial publication bias was observed with Egger’s regression test (intercept: –10.86; *P *< .001), and the pooled effect size with the trim-and-fill method adjusting for imputed missing effects did not change from the pooled effect size. A slightly asymmetrical funnel plot was observed (Figure S6).

**Table 3.** Pooled SMD and Subgroup Analyses of Probiotic for Depression Symptoms

	**No. of studies**	Sample, *n*	SMD (95% CI)	*I^2^*, %	*Q*_(__*df*__)_
Probiotic on depressive symptoms	18	969	−0.96 (−1.31.92, −0.61)	85	
Intervention type^a^					0.08
Adjunctive treatment	12	624	−1.06 (−1.51,-0.60)	86.182	
Stand-alone treatment	5	266	−0.95 (−1.50, −0.41)	77.99	
Diagnosis criteria					1.11
Clinically diagnosed	12	604	−1.09 (−1.54, −0.64)	85.5	
Self-reported/meet threshold criteria	6	365	−0.71 (−1.25, −0.18)	85	
Outcome assessment					1.91
Clinician assessment	7	398	−0.66 (−1.21, −0.11)	86	
Self-assessment	11	571	−1.15 (−1.56, −0.73)	82	
Length of intervention					7.10*
<8 weeks	7	334	−1.54 (−2.18, −0.90)	85	
8 weeks or more	11	635	−0.59 (−0.86, −0.33)	63	
Comorbidities					0.98
With comorbidity	4	213	−1.41 (−2.55, −0.28)	92	
Without comorbidity	14	756	−0.82 (−1.13, −0.51)	76	
Types of probiotics					0.11
Multi-strain probiotic	11	605	−0.92 (−1.46, −0.38)	90	
Single-strain probiotic	7	364	−1.03 (−1.41, −0.65)	66.23	

* *P *≤ .05. *Q* denotes Cochran's *Q* statistic.

a Romjin et al had psychological therapy along with a probiotic—hence, excluded from the subgroup analysis for adjunctive intervention type to stand-alone treatment type.

Abbreviation: SMD, standardized mean difference.

Subgroup analysis indicated varying effect sizes (SMD) for anxiety reduction with probiotics (Table 4; Figures S14–S19). However, a nonsignificant value from Cochran's *Q* statistics showed relatively homogenous effect sizes across the subgroups of studies included in the meta-analysis. The effect size was reduced by 33% in studies after excluding 4 high-risk studies (SMD: –0.39; 95% CI: –0.63, –0.15), with no heterogeneity (*I^2^* = 0%, *P *= .49) (Figure S4). A significant publication bias was observed with Egger’s regression test (intercept: –9.62; *P *= .00), but the pooled effect size with the trim-and-fill method adjusting for imputed missing effects remained unchanged (adjusted SMD = −0.524; 95% CI: −0.701, −0.346). The funnel plot of effect sizes was asymmetrical (Figure S7).

**Table 4.** Pooled SMD and Subgroup Analyses of Probiotic for Anxiety Symptoms

	No. of studies	Sample, *n*	SMD (95% CI)	*I^2^*, %	***Q*_(*df*)_**
Probiotic on anxiety symptoms	9	510	−0.59 (−0.98, −0.19)	79.4	
Intervention type^a^					0.65
Adjunctive treatment	4	205	−0.83 (−1.49, −0.17)	80.7	
Stand-alone treatment	4	226	−0.46 (−1.07, 0.15)	81.1	
Diagnosis criteria					2.68
Clinically diagnosed	3	145	−1.05 (−1.77, −0.33)	76.3	
Self-reported	6	365	−0.37 (−0.76, 0.02)	71.5	
Outcome assessment					1.31
Clinician assessment	3	186	−0.31 (−0.71, 0.08)	45	
Self-assessment	6	324	−0.71 (−1.26, −0.16)	83	
Length of intervention					1.88
Less than 8 weeks	4	215	−0.88 (−1.68, −0.09)	87	
8 weeks and more	5	295	−0.31 (−0.53, −0.08)	0	
Comorbidities					0.92
With comorbidity	2	104	−1.09 (−2.38, 0.19)	89.5	
Without comorbidity	7	406	−0.44 (−0.80, −0.08)	69.6	
Types of probiotics					0.62
Multi-strain	7	406	−0.50 (−0.95, −0.05)	80	
Single-strain	2	104	−0.91 (−1.81, −0.00)	79.95	

*Q* denotes Cochran's *Q* statistic.

a Romjin et al had psychological therapy along with a probiotic—hence, excluded from the subgroup analysis for adjunctive intervention type to stand-alone treatment type.

Abbreviation: SMD, standardized mean difference.

### Certainty of Evidence (GRADE Criteria)

The quality and certainty of evidence of probiotic interventions were evaluated based on GRADE criteria for the primary outcomes of depression and anxiety severity and was assessed as discussed in the following sections.

#### Effectiveness of Probiotics for Depression

Certainty in the effect estimates of probiotic interventions for depression was rated as low. Eighteen RCTs with some limitations in study design (eg, non-blinding) showed consistent results with moderately wide CIs. The certainty of evidence was downgraded 2 levels due to substantial heterogeneity (*I^2^* = 85%) that could not be fully explained by comparator type or outcome assessment method; concerns regarding 7 studies showing high risk of bias in multiple RCTs, which resulted in a 32% reduction in pooled effect size; imprecision; slightly asymmetrical funnel plot; and existence of substantial publication bias. Therefore, the certainty of evidence in the effect estimate was downgraded 2 levels.

#### Effectiveness of Probiotics on Anxiety

Certainty in the effect estimates for probiotic interventions for anxiety was rated as moderate. Nine RCTs showed narrow CIs and consistency in findings. However, the evidence was downgraded 1 level due to concerns regarding risk of bias, high heterogeneity (*I^2^* = 79%), slight funnel plot asymmetry, and publication bias.

## DISCUSSION

This meta-analytic review comprehensively examines the effectiveness of prebiotics, probiotics, and synbiotics in alleviating depression and anxiety in clinically diagnosed samples exclusively. Overall, our meta-analytic findings suggested that probiotics had large effect size reductions in depression (SMD: –0.96; 95% CI: –1.31, –0.61) and moderate reductions in anxiety symptoms (SMD: –0.59; 95% CI: –0.98, –0.19) compared with the control group. Prebiotics showed a nonsignificant trend towards reducing depression, but the small number of studies in the analysis limits interpretability. In probiotic interventions, subgroup analyses suggested that studies with shorter durations (<8 weeks) had larger effects on depression, while studies using single-strain probiotics had larger effects on anxiety.

Results of 3 studies revealed nonsignificant effects of prebiotics on depression symptoms that are consistent with other meta-analyses.^20^^,^^25^^,^^26^^,^^53^ Although it has been proven in animal models that prebiotics can alleviate depressive-like behavior,^54^ data from clinical studies are still scarce and meta-analytic data found no clear evidence for depression. Future large-scale clinical trials to reveal the longitudinal effectiveness and dosage of prebiotics that would yield an effect on depression and anxiety in clinically diagnosed samples are recommended.

Despite no clear benefits of prebiotics on depression and anxiety, the findings from this review demonstrated strong effectiveness of probiotics in reducing depression and anxiety symptoms, which are consistent with other meta-analyses. Liu et al^26^ found that, while no difference between prebiotics and control conditions in reducing depressive symptom scores was observed, probiotics exerted significant antidepressant effects.

The effectiveness of probiotics significantly reduced depression and anxiety when patients were diagnosed clinically by following standard methods (ICD/DSM criteria) compared with diagnosis from self-report. The effectiveness was also larger in trials with a single-strain probiotic intervention, with shorter durations, and in samples with other medical conditions.

This larger effect size in reducing depression and anxiety in a clinically diagnosed group (ICD/DSM criteria) is consistent with subgroups focusing on clinical samples compared with community samples in previous meta-analyses.^5–8^ This study is the first to show significant efficacy in reducing anxiety symptoms by probiotic interventions. The higher effectiveness of probiotic interventions in clinically diagnosed individuals with depression and anxiety can be attributed to factors such as precise diagnosis and increased homogeneity in the study population, greater symptom severity, consistent baseline characteristics to detect significant improvement from the intervention, potential comorbidities, and an enhanced placebo effect due to heightened motivation and expectation for symptom improvement. This highlights the importance of diagnostic context in interpreting intervention outcomes and suggests that probiotics may be combined with pharmacotherapy for a more personalized approach to treat varying depression and anxiety symptoms in clinically diagnosed patient populations. However, contrasting results were observed when the score of outcomes was assessed by clinician vs self-assessment. Clinician assessment showed a lower effect size compared with studies where patients reported the score in questionnaires by themselves, and this may be explained by overestimation and social desirability bias. Future studies would benefit from including an objective measure of gut-microbiome composition alongside self-reported and clinician-administered assessments to increase reliability.

Interventions of up to 8 weeks duration were more effective compared with those of 8 weeks or more, which aligns with a meta-analysis of 21 trials investigating probiotic effects on irritable bowel syndrome (IBS).^9^ However, 3 shorter-duration trials,^10–12^ characterized by higher effect sizes, may be influenced by a high risk of bias, potentially leading to overestimation of results. Longer intervention durations may be associated with a higher dropout rate or a plateau effect, whereas shorter durations could ensure better compliance and treatment response. Among the trials, 7 reported over 90% compliance within 8 weeks, while a 90-day trial had a 60% compliance rate.

Single-strain probiotics have shown greater effectiveness compared with multi-strain alternatives, consistent with meta-analysis findings in IBS treatment.^9^ One possible explanation is that the potential competition among the ingested strains could result in adverse interactions, despite the diverse benefits offered by multi-strain probiotics.^13^ Additionally, it is difficult to ascertain whether the effectiveness is from the specific strain in the mixture or from the interaction.^14^ However, limited trials directly comparing single- and multi-strain effectiveness, coupled with methodological and compositional variations, hinder definitive conclusions.^14^ In the conducted meta-analysis, within the single-strain probiotics subgroup, greater effectiveness was associated with higher dosages of specific species (*Bacillus coagulans*, *Clostridium butyricum*, *Lactobacillus*, and *Bifidobacterium)*. This highlights how species selection in probiotic formulations plays a crucial role, as specific species might have a stronger influence on outcomes than sheer species quantity.

Substantially larger effect sizes in reducing both depression and anxiety scores were observed in 4 studies where patients had other comorbidities. This is likely attributed to the comorbid conditions in this study being linked to gut health (eg, IBS, small intestinal bacterial overgrowth, and constipation), where probiotics are intended to exert a beneficial impact in reducing gastrointestinal conditions, making the results unsurprising.^15^ Given the GBA, these improvements might have indirectly influenced the changes in depression and anxiety scores. As a result, the studies that included comorbid conditions need to be interpreted cautiously. It remains unclear whether the observed reduction in depression and anxiety is primarily due to the alleviation of the medical conditions treated with probiotics or if it is a result of potential improvements in the patients’ comorbid conditions.

The lack of significant differences between probiotics-only and probiotics-plus-antidepressant groups in reducing depression and anxiety has important implications for treatment trajectories across the depression disease pathway. Since probiotics alone or alongside antidepressant medication yield similar outcomes, this suggests a possible treatment option for those who may be exploring tapering off antidepressant medication, or who may prefer a more cost-effective or natural alternative option. Additionally, many patients experience undesirable side effects (eg, gastrointestinal discomfort, weight gain, gut inflammation) from traditional antidepressant medication due to the inhibitory effect on the growth of microbiota strains.^16^^,^^17^ The add-on of probiotics may lead to synergistic effects, potentially enhancing treatment efficacy without increased side effects in patients who take antidepressants regularly.^16^^,^^17^ Not all individuals respond to antidepressant medications in the same way, and the findings from this review suggest that probiotics are an additional effective treatment option, with or without medication, that can be tailored to individual needs.

As there was only 1 paper that reported on this, the combined efficacy of prebiotics and probiotics (synbiotics) could not be substantiated. However, the single study reported improved depressive scores in clinically diagnosed patients with antidepressant treatment. Future multi-arm trials would benefit from examining synbiotic supplementation compared with pre- or probiotics separately to establish treatment effectiveness.

The main strengths of this systematic review and meta-analysis were the inclusion of 21 clinical trials, comprehensive subgroup analysis, focusing on clinically diagnosed samples, and addressing the heterogeneity after excluding high-risk studies in sensitivity analysis. This represents nearly double the RCTs reviewed by Zhang et al^20^ and adds to the literature pertaining to adjunctive treatment approaches for psychiatric disorders. In particular, the comprehensive subgroup analyses exploring multiple confounding factors (probiotic type, intervention duration, comorbidities) provided additional valuable insights into treatment effects across subgroups, detection of effect modification, and informing personalized treatment strategies for adults with mental health disorders.

Despite the strengths of this study, the findings must be interpreted with caution. High heterogeneity across studies, varying in design, intervention type, dosage, and settings, hinders generalizability. Most studies were underpowered and had risk of bias concerns. Diverse age ranges and predominantly female gender across trials present additional limitations, as gut microbiome and treatment response differ based on age and gender.^55^^,^^18^^,^^19^ Global variations in environmental factors, genetics, and diet may have also influenced treatment responses.^18^ Additionally, the severity and subtypes of depression and anxiety could not be explored, which may affect generalizability. Depression and anxiety have different prognoses between genders, which could not be explored.^20^ Differing assessment tools (eg, Beck Depression Inventory [BDI], the 9-item Patient Health Questionnaire [PHQ-9], Montgomery–Åsberg Depression Rating Scale [MADRS]) may have influenced the pooled effect of meta-analysis, even after standardization, as different tools address different aspects of depression and anxiety. The variation in clinical diagnostic criteria and use of different methods (eg, ICD, DSM, BDI) and cutoff values to identify depression severity and subtype introduced additional heterogeneity. Future trials would benefit from addressing these limitations by following targeted recruitment strategies aimed at larger, homogenous samples, and embedding objective measures of gut microbiota composition (eg, stool samples) in addition to self-reported or clinician-diagnosed measures to establish more precise treatment trajectories.

Overall, this meta-analytic review provides important clinical implications for the use of prebiotics and probiotics in the treatment of depression and anxiety in clinical populations. Larger-scale investigations involving diverse ethnic populations are needed to establish optimal probiotic treatment effectiveness, including dose–response relationships. Standardized methods for assessing psychiatric scores and the inclusion of personalized interventions that diagnose, monitor, and treat the gut microbiome alongside mental health treatment hold promise for clinical practice.

## CONCLUSION

This systematic review and meta-analysis comprehensively assessed the effectiveness of prebiotics and prebiotics on depression and anxiety symptom severity in clinically diagnosed adults. Probiotic interventions, either as a stand-alone treatment or combined with antidepressant medication use, show effectiveness in reducing symptoms in clinically diagnosed patients, with single-strain probiotics showing the largest effects. Comorbidities appear to impact probiotics' mental health effects. Further research into probiotic effectiveness across various demographic factors, such as ethnicity, age, gender, and mental health diagnoses, with and without comorbid clinical conditions, is recommended.

## Supplementary Material

nuae177_Supplementary_Data

## Data Availability

No data are available.
