# Supplementary material for: Effects of Prebiotics and Probiotics on Symptoms of Depression and Anxiety in Clinically Diagnosed Samples: Systematic Review and Meta-analysis of Randomized Controlled Trials
Source: Nutr Rev. 2024 Dec 28;83(7):e1504–20. doi: 10.1093/nutrit/nuae177 (PMC12166186; doi:10.1093/nutrit/nuae177)
Supplement: nuae177_Supplementary_Data [file nuae177_supplementary_data.docx]

# **Appendix S1**

This appendix contains figures showing risk of bias, forest plots after conducting sensitivity analysis excluding high risk of bias papers, funnel plots, and subgroup analysis of probiotic and depression, and subgroup analysis of probiotic and anxiety. A table with search strategy and results is also added.

# **Supplementary 1: Summarized risk of bias**


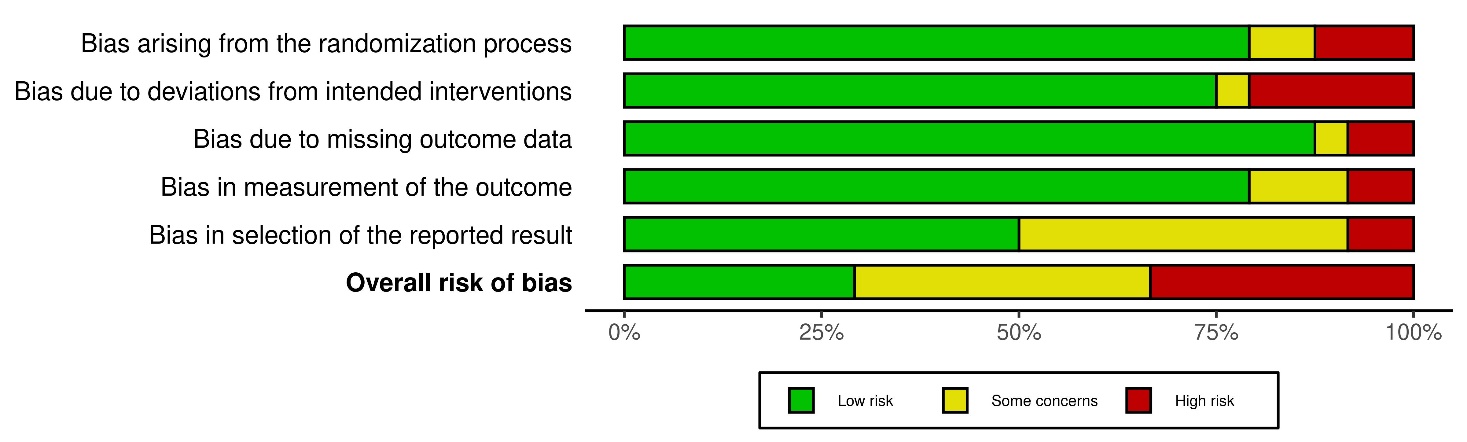


Figure S1: Bar plot of risk of bias with individual domains.

# **
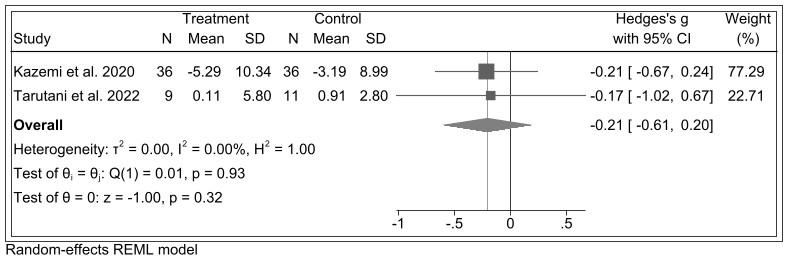
****Supplementary 2: Sensitivity analysis**

Figure S2: Forest plot of prebiotic effect on depression excluding high risk paper.

**
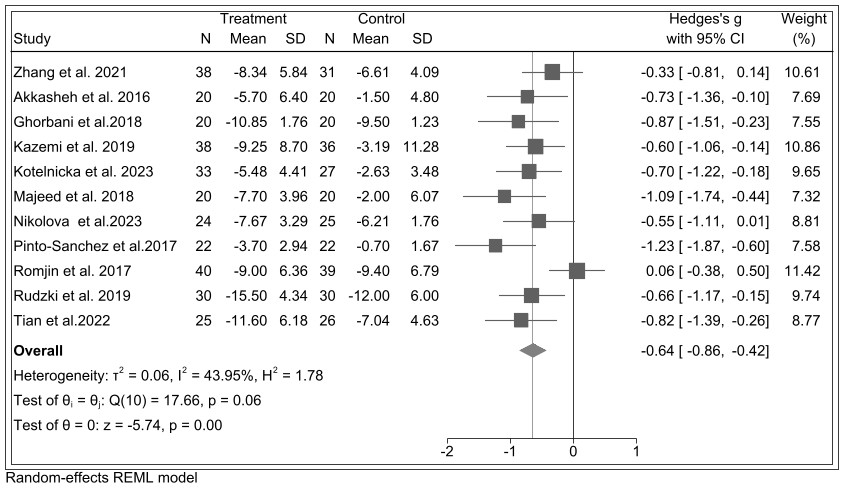
**

Figure S3: Forest plot of probiotic effect on depression excluding high risk paper.

**
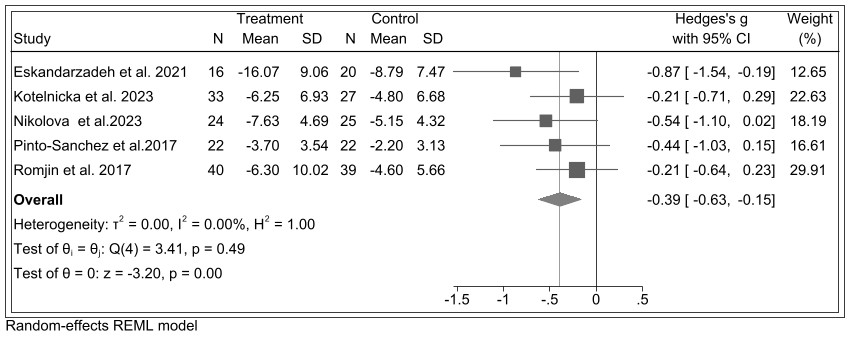
**

Figure S4: Forest plot of probiotic effect on anxiety excluding high risk paper.

**Supplementary 3: Funnel plot**


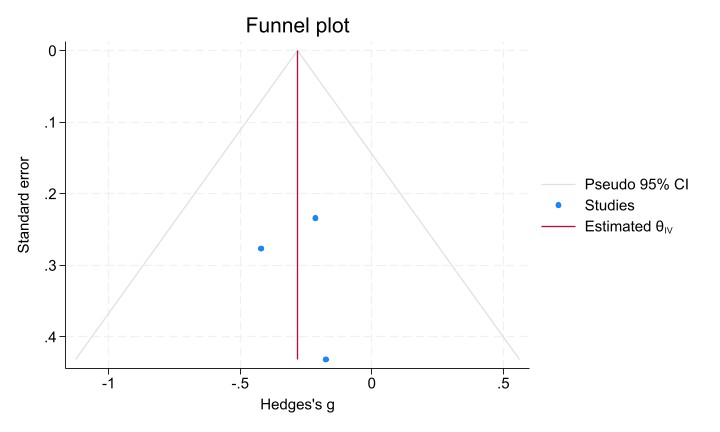


Figure S5: Funnel plot of studies assessing prebiotic and depression


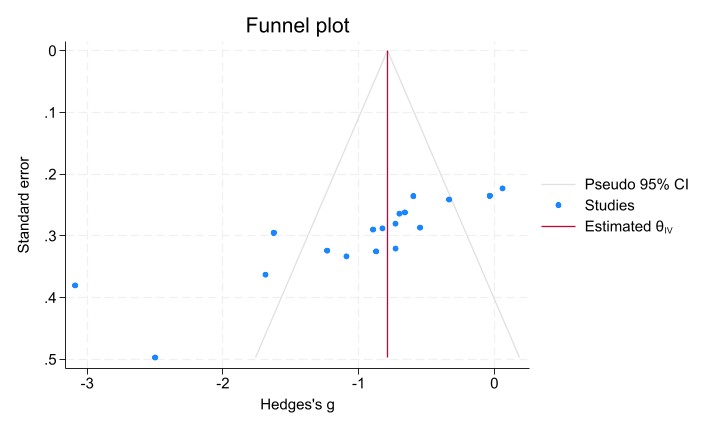


Figure S6: Funnel plot of studies assessing probiotic and depression


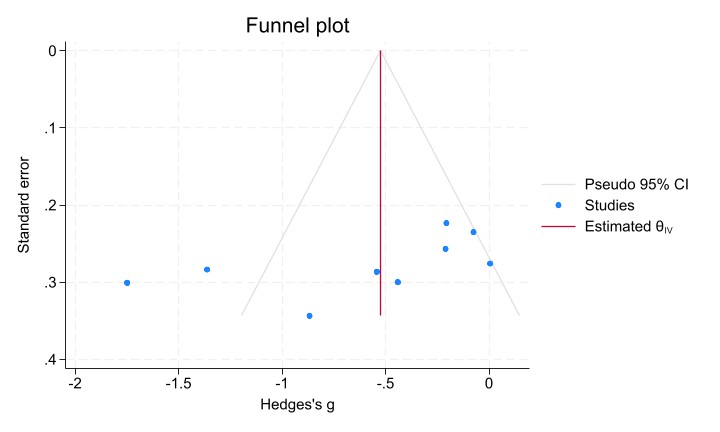


Figure S7: Funnel plot of studies assessing probiotic and anxiety

# **Supplementary 4: Subgroup analyses of Probiotic and Depression**

#
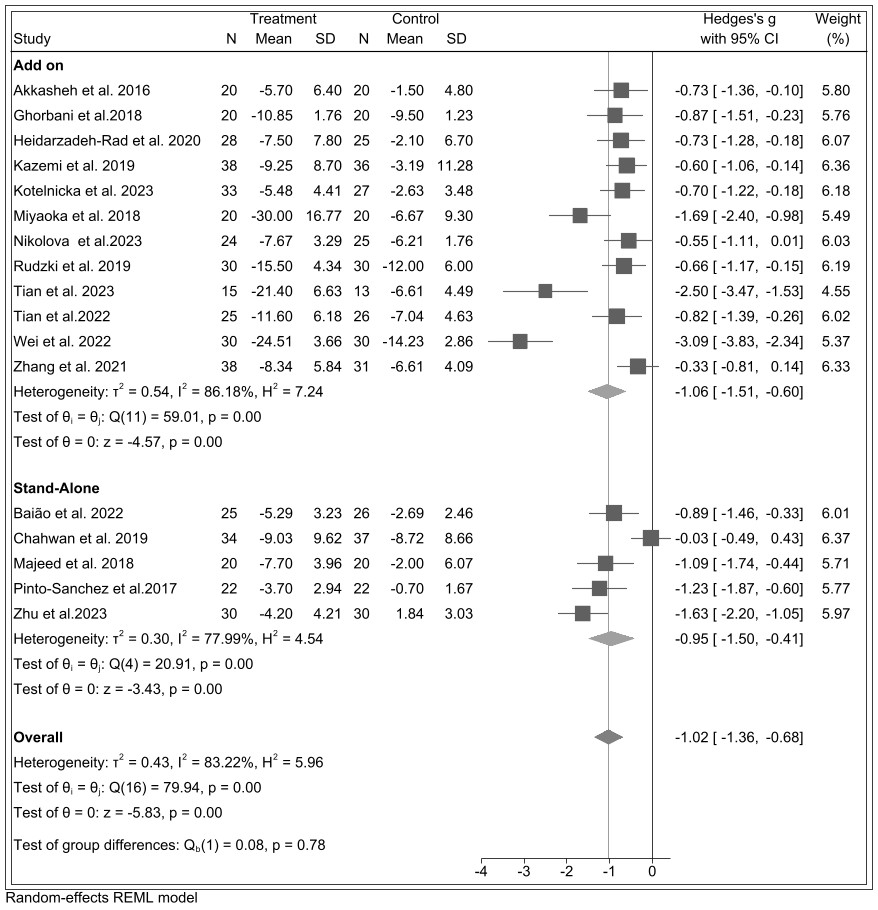


Figure S8: Forest plot of probiotic reducing depression grouped by treatment type (adjunctive vs. stand-alone)


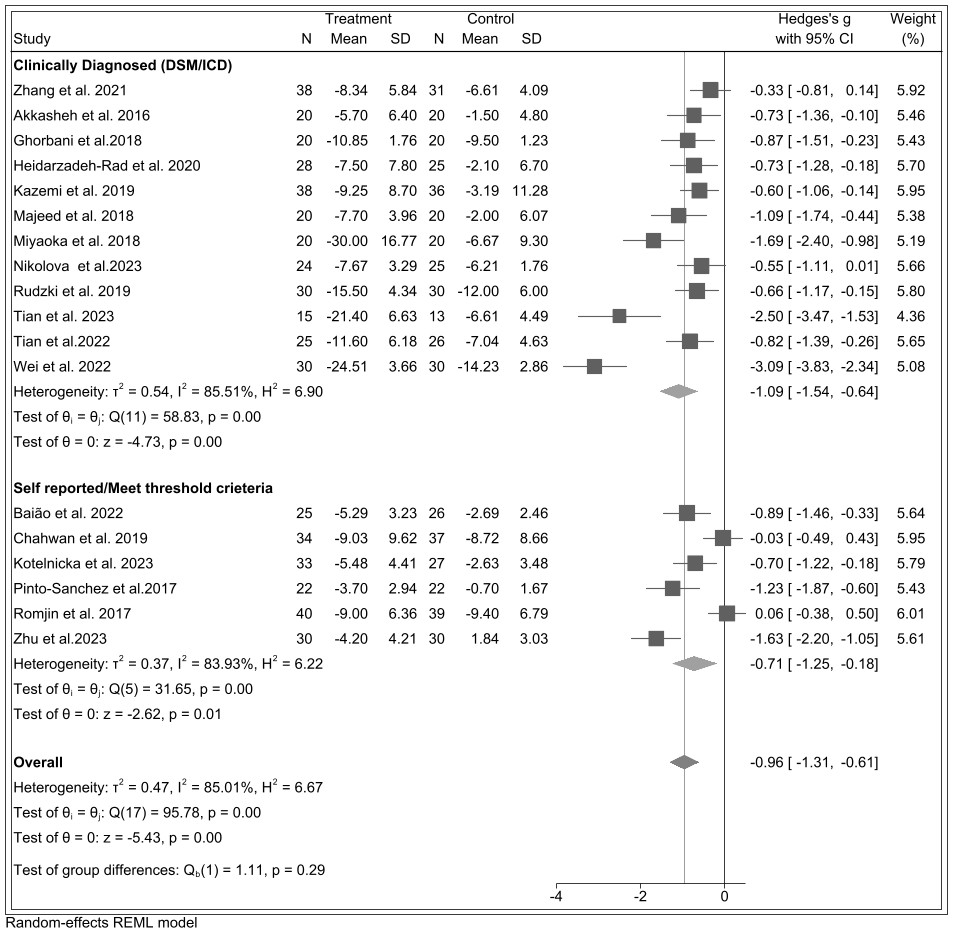


Figure S9: Forest plot of probiotic reducing depression grouped by diagnosis method (Clinical vs self-report)

**
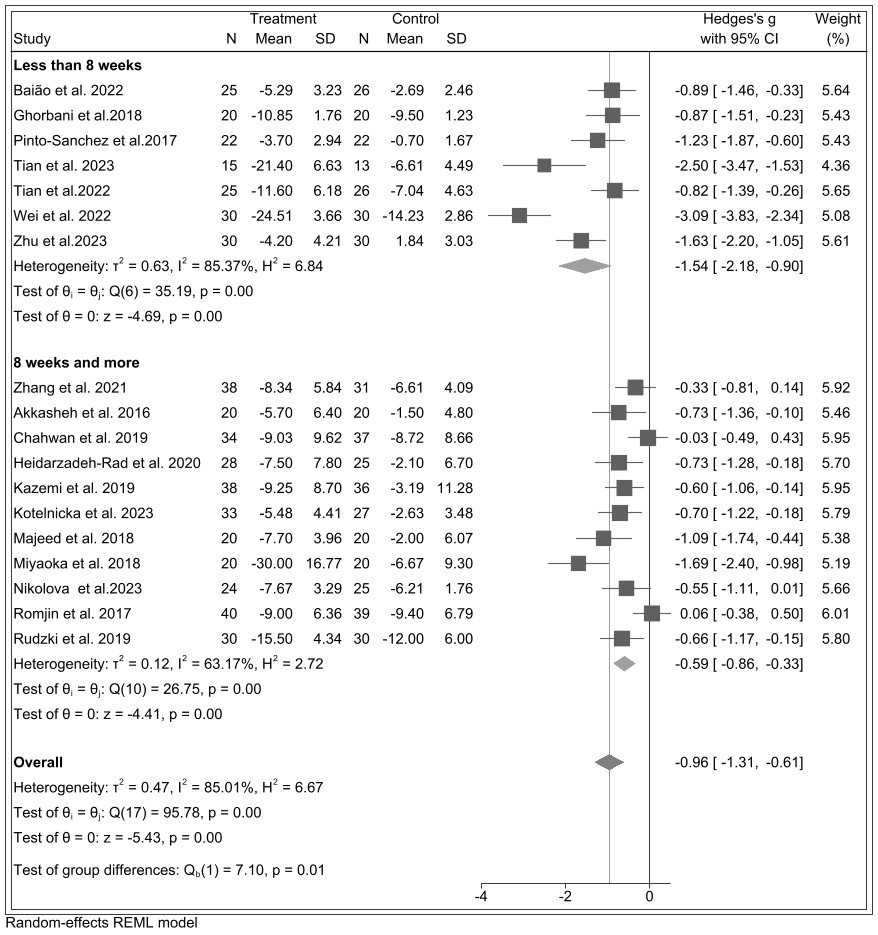
**

Figure S10: Forest plot of probiotic reducing depression grouped by duration (<8 weeks vs ≥ 8 weeks)


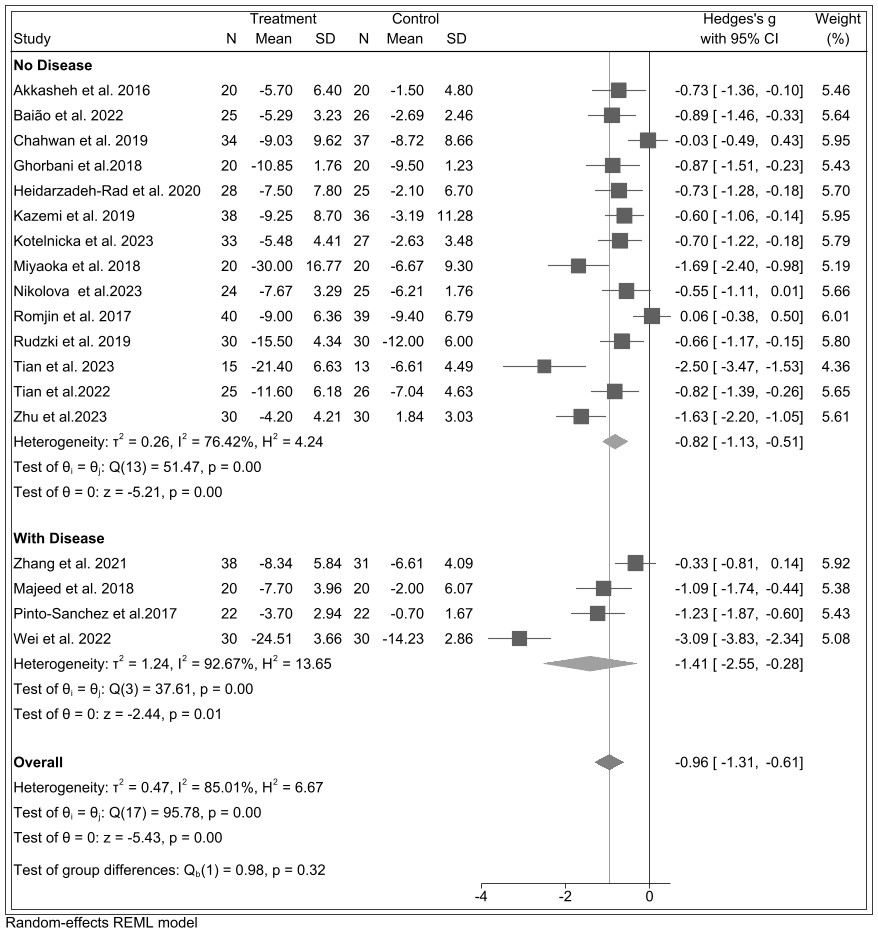


1. Probiotic effect in depression (clinician assessed outcome vs self-assessed outcome)

Figure S11: Forest plot of probiotic reducing depression grouped by comorbidities (with disease vs no disease)


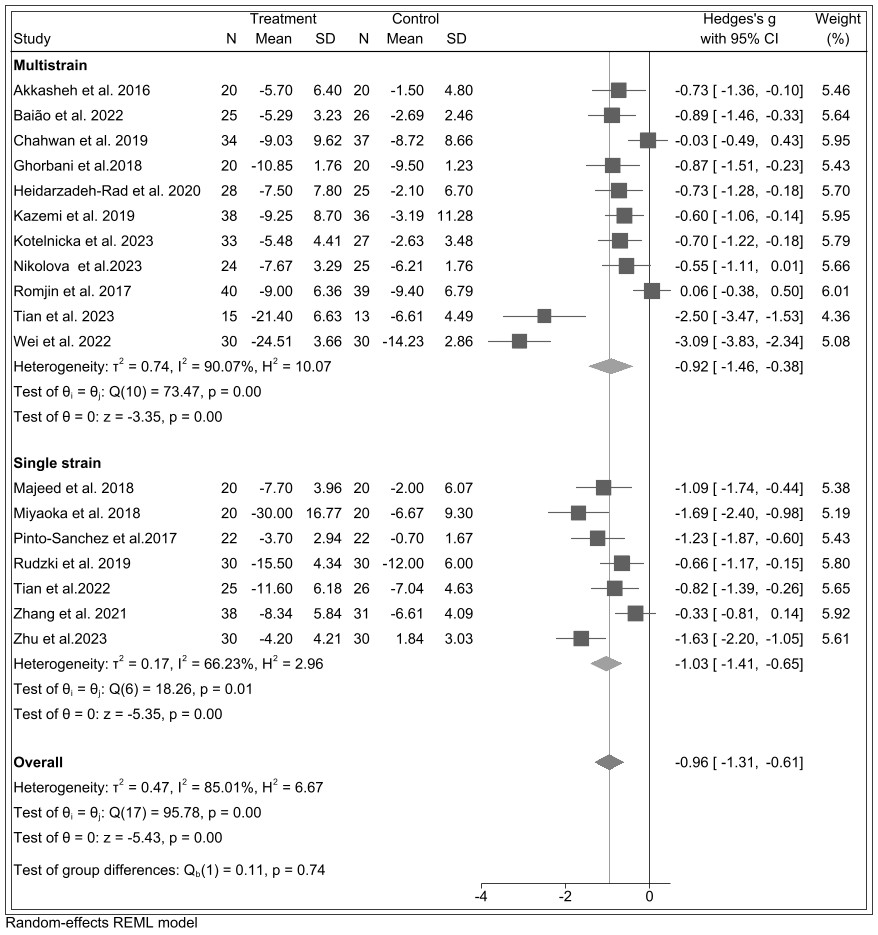


Figure S12: Forest plot of probiotic reducing depression grouped by probiotic form (Multi strain vs Single strain)


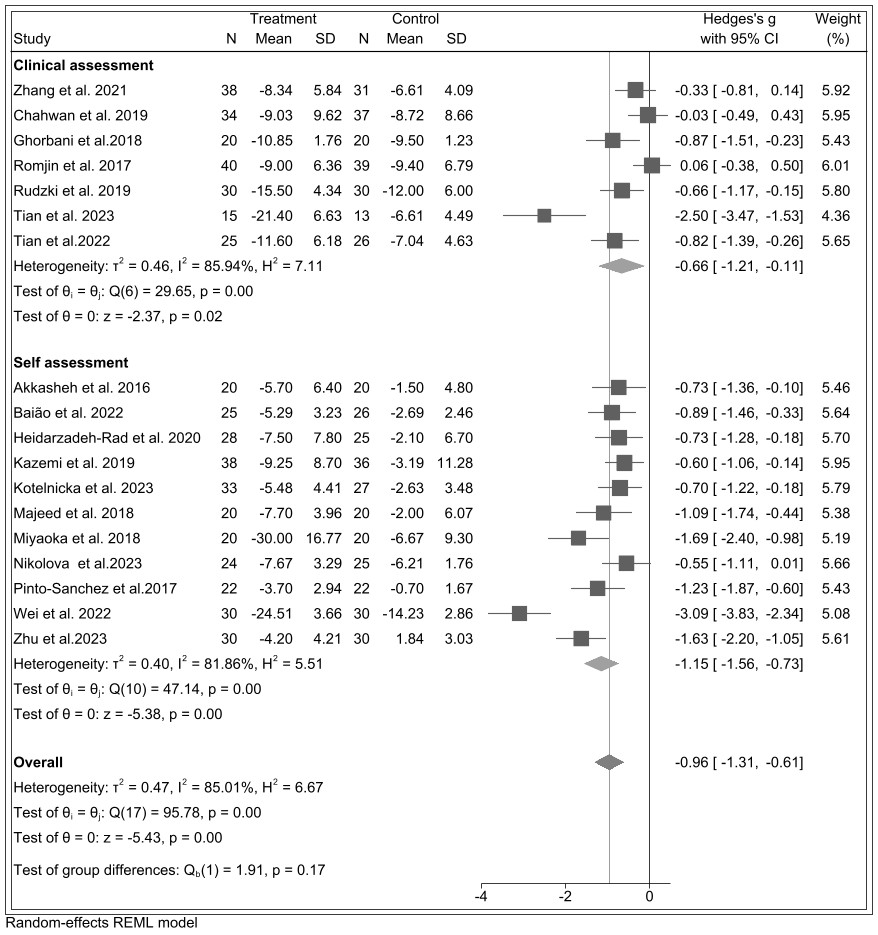


Figure S13: Forest plot of probiotic reducing depression grouped by assessment method (clinician vs self-assessment)

# Supplementary 4: Subgroup analyses of Probiotic and Anxiety


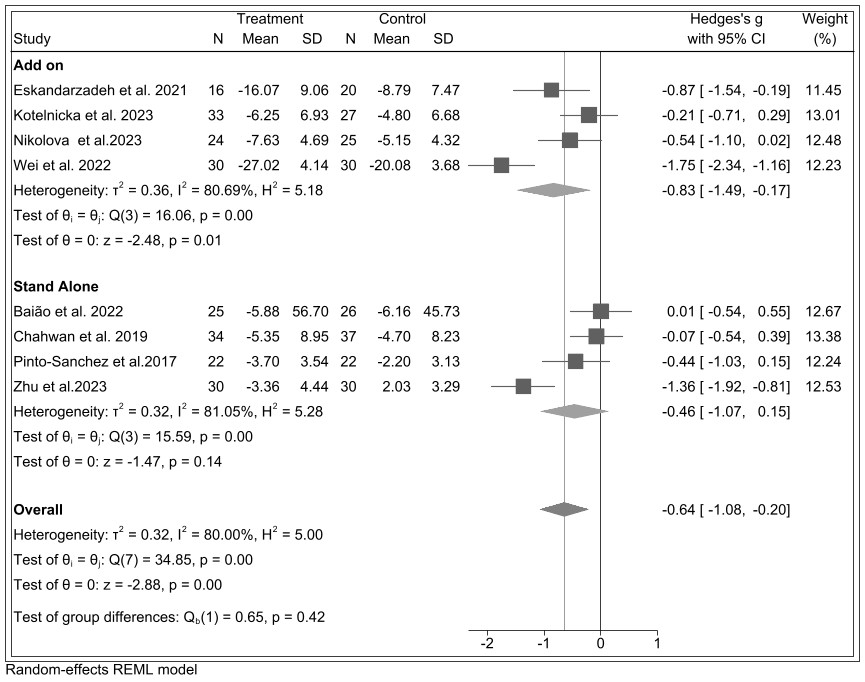


Figure S14: Forest plot of probiotic reducing anxiety grouped by treatment type (adjunctive vs. stand-alone)


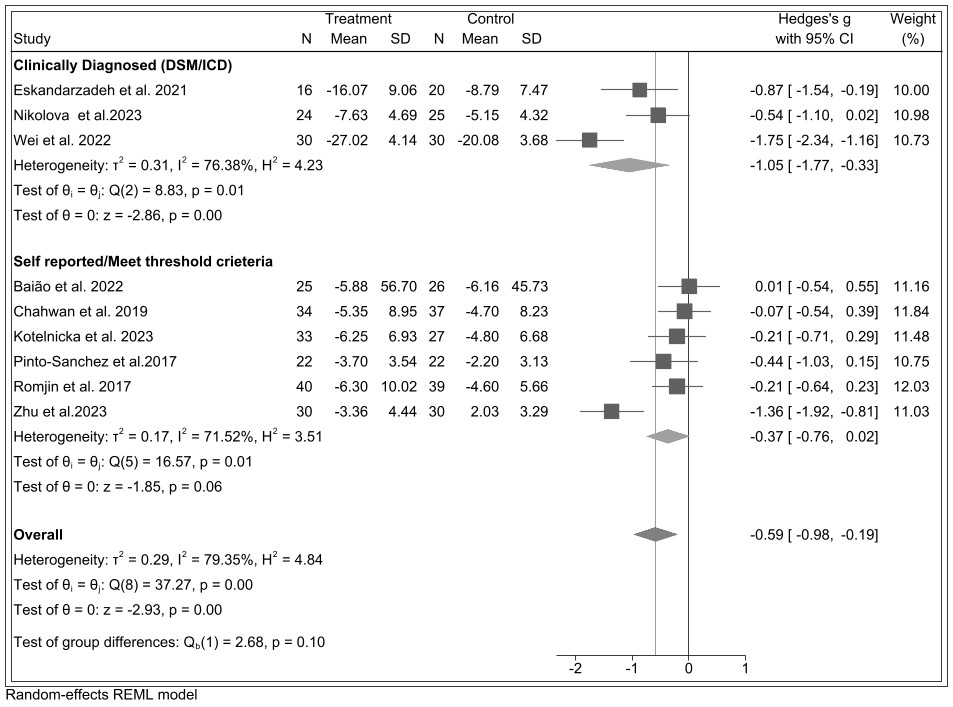


Figure S15: Forest plot of probiotic reducing anxiety grouped by diagnosis method (Clinical vs self-report)


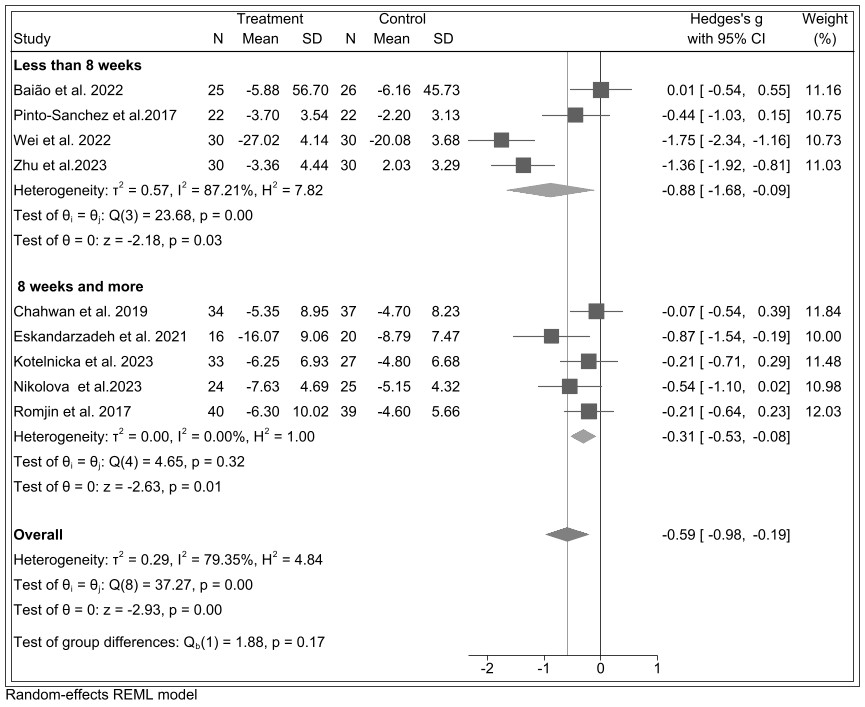


Figure S16: Forest plot of probiotic reducing anxiety grouped by duration (>8 weeks vs ≥8 weeks)


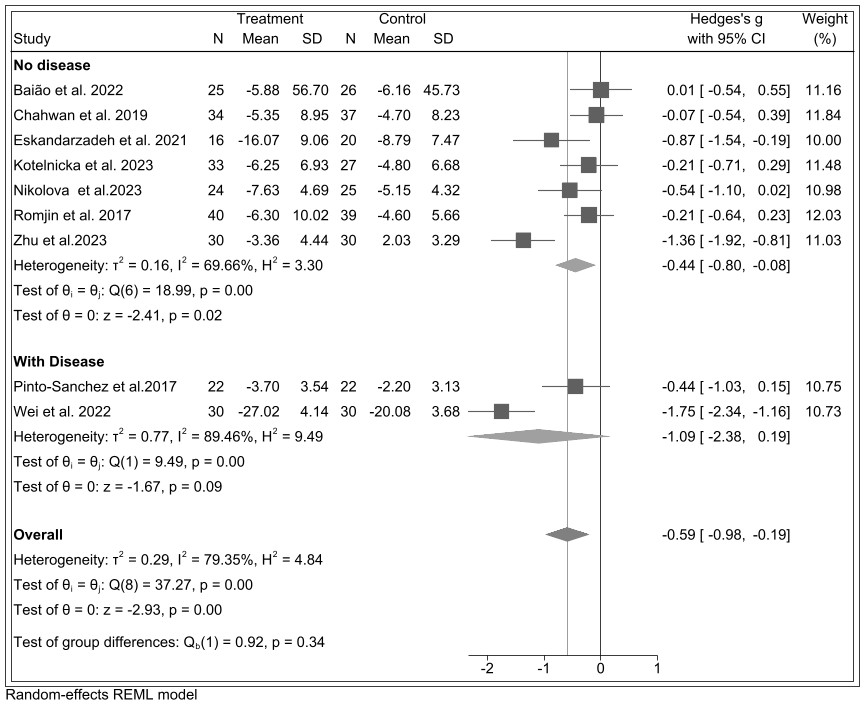


Figure S17: Forest plot of probiotic reducing anxiety grouped by comorbidities (with disease vs no disease)


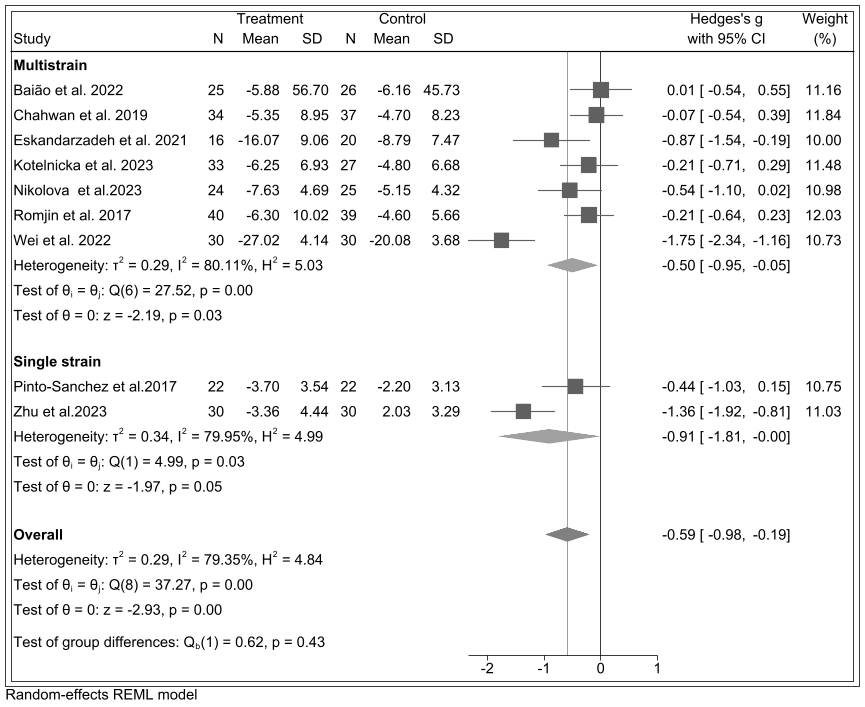


Figure S18: Forest plot of probiotic reducing anxiety grouped by probiotic form (Multi strain vs Single strain)


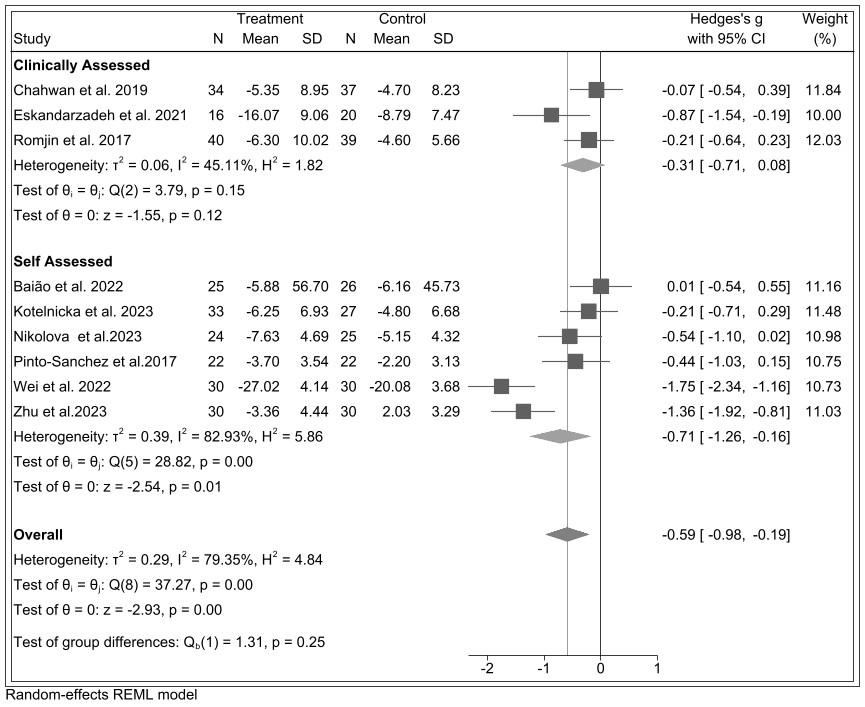


Figure S19: Forest plot of probiotic reducing anxiety grouped by assessment method (clinician vs self-assessment)

# **Table S1: Search strategy in databases**

|  | **DATABASE** | **Results** |
| --- | --- | --- |
| Number | [**Medline) 1946 to present**](https://ovidsp.ovid.com/ovidweb.cgi?T=JS&NEWS=N&PAGE=main&SHAREDSEARCHID=6wLTbOaLVcvQ9E4TVldSQYZuB4CRKq39kCESBpC71rZGXQzCbU5bwxtRl4LLE5sK7) |  |
| 1 | exp Depressive Disorder/ | 121446 |
| 2 | exp Anxiety Disorders/ | 89985 |
| 3 | exp "Schizophrenia Spectrum and Other Psychotic Disorders"/ | 162370 |
| 4 | anxiety/ or depression/ | 217971 |
| 5 | Mental Disorders/ | 177193 |
| 6 | (depress* or mood? or dysthymi* or anxiet* or anxious or phobia? or psychos?s or psychotic? or schizophren*).ti,ab,kf. | 893363 |
| 7 | (((obsessive compulsive or panic or phobic or stress) adj2 disorder?) or (post-traumatic stress or post-traumatic distress or posttraumatic stress or posttraumatic distress or ptsd or ptdd)).ti,ab,kf. | 78185 |
| 8 | ((mental* or psycholog* or psychiatric) adj2 (health or ill* or disorder?)).ti,kf. | 157240 |
| 9 | 1 or 2 or 3 or 4 or 5 or 6 or 7 or 8 | 1218925 |
| 10 | prebiotics/ or probiotics/ or synbiotics/ | 26681 |
| 11 | exp lactobacillus/ or exp lactococcus/ or streptococcus salivarius/ or streptococcus thermophilus/ | 30328 |
| 12 | Bacillus coagulans/ | 176 |
| 13 | bifidobacterium/ or bifidobacterium animalis/ or bifidobacterium bifidum/ or bifidobacterium breve/ or exp bifidobacterium longum/ | 7150 |
| 14 | Clostridium butyricum/ | 372 |
| 15 | Faecalibacterium prausnitzii/ | 133 |
| 16 | (prebiotic? or pre-biotic? or probiotic? or pro-biotic? or synbiotic? or syn-biotic?).ti,ab,kf. | 46500 |
| 17 | (((B or Bacillus) adj coagulans) or B-GOS or bifidobacteria or ((B or Bifidobacterium) adj (animalis or bifidium or bifidus or breve or lactis or longum)) or Bimuno or galactooligosaccharide or ((C or Clostridium) adj butyricum) or ((F or faecalibacterium) adj prausnitzii) or FOS or fructooligosaccharide or GOS or inulin or ((l or lactobacillus) adj (plantarum or acidofilus or acidophilus or brevis or Bulgari* or cas?ei or fermentum or gasseri or helveticus or paraca?sei or pentosus or reuteri or rhamnosus or salivarius or delbrueckii)) or ((l or lactococcus) adj lactis) or ((S or saccharomyces) adj boulardii) or ((S or Streptococ?us) adj (salivarius or thermophiles or thermophilus)) or XOS).ti,ab,kf. | 88626 |
| 18 | (Actinobacteria or Akkermansia or Atopobium or Bacteroidaceae or Bacteroides or Bacteroidetes or Bifidobacteriaceae or Bifidobacterium or Clostridiaceae or Clostridium or Corynebacterium or Dialister or Enterobacteriaceae or Enterococcus or Escherichia or Euryarchaeota or Faecalibacterium or Firmicutes or Fusobacteria or Lachnospiraceae or Lactobacillus or Methanobrevibacter or Oscillospira or Peptostreptococcaceae or Prevotella or Prevotellaceae or Propionibacterium or Proteobacteria or Rikenellaceae or Ruminococcaceae or Ruminococcus or Sneathia or Staphylococcus or Streptococcus or Veillonella or Verrucomicrobia).ti,kf. | 310223 |
| 19 | Gastrointestinal Microbiome/ and supplement*.mp. | 4358 |
| 20 | (((gut or gastrointestin* or gi or intestinal) adj (microb* or flora)) and supplement*).ti,ab,kf. | 7785 |
| 21 | 10 or 11 or 12 or 13 or 14 or 15 or 16 or 17 or 18 or 19 or 20 | 417340 |
| 22 | 9 and 21 | 4739 |
| 23 | randomized controlled trial.pt. | 592992 |
| 24 | controlled clinical trial.pt. | 95309 |
| 25 | randomized.ab. | 603684 |
| 26 | placebo.ab. | 238282 |
| 27 | drug therapy.fs. | 2591640 |
| 28 | randomly.ab. | 408575 |
| 29 | trial.ab. | 648761 |
| 30 | groups.ab. | 2518483 |
| 31 | 23 or 24 or 25 or 26 or 27 or 28 or 29 or 30 | 5660024 |
| 32 | exp animals/ not humans.sh. | 5122628 |
| 33 | (rat or rats or mouse or mice or murine or rodent?).ti. | 1536669 |
| 34 | 32 or 33 | 5390568 |
| 35 | 31 not 34 | 4879921 |
| 36 | 22 and 35 | 679 |
| **Number** | [**Embase 1974 to present**](https://ovidsp.ovid.com/ovidweb.cgi?T=JS&NEWS=N&PAGE=main&SHAREDSEARCHID=jn1INo1qXWqfATjjAK15S3917xOtvxOeg6sCZeEXXRlfxVq3H5lWxVAAOBVTXvHc) | **Results** |
| 1 | exp *depression/ or *mood disorder/ | 278454 |
| 2 | *anxiety/ or exp *anxiety disorder/ | 192369 |
| 3 | exp *psychosis/ | 188347 |
| 4 | *mental disease/ | 114150 |
| 5 | mental patient/ | 29310 |
| 6 | (depress* or mood? or dysthymi* or anxiet* or anxious or phobia? or psychos?s or psychotic? or schizophren*).ti,ab,kf. | 1204363 |
| 7 | (((obsessive compulsive or panic or phobic or stress) adj2 disorder?) or (post-traumatic stress or post-traumatic distress or posttraumatic stress or posttraumatic distress or ptsd or ptdd)).ti,ab,kf. | 104549 |
| 8 | ((mental* or psycholog* or psychiatric) adj2 (health or ill* or disorder?)).ti,kf. | 171679 |
| 9 | 1 or 2 or 3 or 4 or 5 or 6 or 7 or 8 | 1502384 |
| 10 | microbial products not classified elsewhere/ or bifidobacterin/ or bifidobacterium breve extract/ or corynebacterium parvum extract/ or lactobacillus casei extract/ or exp probiotic agent/ or streptococcus thermophilus extract/ or prebiotic agent/ or synbiotic agent/ | 60351 |
| 11 | exp Lactobacillus/ or exp lactococcus/ or streptococcus salivarius/ or streptococcus thermophilus/ | 71935 |
| 12 | Bacillus coagulans/ | 920 |
| 13 | exp bifidobacterium/ | 20889 |
| 14 | Clostridium butyricum/ | 1542 |
| 15 | Faecalibacterium prausnitzii/ | 1922 |
| 16 | (prebiotic? or pre-biotic? or probiotic? or pro-biotic? or synbiotic? or syn-biotic?).ti,ab,kf. | 58207 |
| 17 | (((B or Bacillus) adj coagulans) or B-GOS or bifidobacteria or ((B or Bifidobacterium) adj (animalis or bifidium or bifidus or breve or lactis or longum)) or Bimuno or galactooligosaccharide or ((C or Clostridium) adj butyricum) or ((F or faecalibacterium) adj prausnitzii) or FOS or fructooligosaccharide or GOS or inulin or ((l or lactobacillus) adj (plantarum or acidofilus or acidophilus or brevis or Bulgari* or cas?ei or fermentum or gasseri or helveticus or paraca?sei or pentosus or reuteri or rhamnosus or salivarius or delbrueckii)) or ((l or lactococcus) adj lactis) or ((S or saccharomyces) adj boulardii) or ((S or Streptococ?us) adj (salivarius or thermophiles or thermophilus)) or XOS).ti,ab,kf. | 105561 |
| 18 | (Actinobacteria or Akkermansia or Atopobium or Bacteroidaceae or Bacteroides or Bacteroidetes or Bifidobacteriaceae or Bifidobacterium or Clostridiaceae or Clostridium or Corynebacterium or Dialister or Enterobacteriaceae or Enterococcus or Escherichia or Euryarchaeota or Faecalibacterium or Firmicutes or Fusobacteria or Lachnospiraceae or Lactobacillus or Methanobrevibacter or Oscillospira or Peptostreptococcaceae or Prevotella or Prevotellaceae or Propionibacterium or Proteobacteria or Rikenellaceae or Ruminococcaceae or Ruminococcus or Sneathia or Staphylococcus or Streptococcus or Veillonella or Verrucomicrobia).ti,kf. | 336522 |
| 19 | intestine flora/ and supplement*.mp. | 10687 |
| 20 | (((gut or gastrointestin* or gi or intestinal) adj (microb* or flora)) and supplement*).ti,ab,kf. | 9256 |
| 21 | 10 or 11 or 12 or 13 or 14 or 15 or 16 or 17 or 18 or 19 or 20 | 492119 |
| 22 | 9 and 21 | 7022 |
| 23 | randomized controlled trial/ | 784768 |
| 24 | controlled clinical trial/ | 469240 |
| 25 | random*.ti,ab. | 1966058 |
|  | randomization/ | 99098 |
|  | intermethod comparison/ | 297181 |
|  | placebo.ti,ab. | 365873 |
|  | (Compare or compared or comparison).ti. | 603425 |
|  | ((evaluated or evaluate or evaluating or assessed or assess) and (compare or compared or comparing or comparison)). ab. | 2762777 |
|  | (open adj label).ti,ab. | 108880 |
|  | ((double or single or double or singly) adj (blind or blinded or blindly)).ti,ab. | 274238 |
|  | double blind procedure/ | 210393 |
|  | parallel group*1. ti,ab. | 32173 |
|  | (crossover or cross over).ti,ab. | 124419 |
|  | ((assign$ or match or matched or allocation) adj5 (alternate or group$1 or intervention$1 or patient$1 or subject$1 or participant$1)).ti,ab. | 414653 |
|  | (assigned or allocated).ti,ab. | 488479 |
|  | (controlled adj7 (study or design or trial)).ti,ab. | 450346 |
|  | (volunteer or volunteers).ti,ab. | 282106 |
|  | human experiment/ | 649539 |
|  | [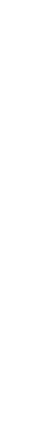](https://ezproxy-prd.bodleian.ox.ac.uk:2483/ovid-a/ovidweb.cgi?&S=DGLOFPGFPGEBIFGIJPPJBHBFJPFIAA00&R=30&Search+Annotations+Options=SA)trial.ti. | 402496 |
|  | 23 or 24 or 25 or 26 or 27 or 28 or 29 or 30 or 31 or 32 or 33 or 34 or 35 or 36 or 37 or 38 or 39 or 40 or 41 | 6303972 |
|  | (rat or rats or mouse or mice or swine or porcine or murine or sheep or lambs or pigs or piglets or rabbit or rabbits or cat or cats or dog or dogs or cattle or bovine or monkey or monkeys or trout or marmoset$1).ti. and animal experiment/ | 1226123 |
|  | Animal experiment/ not (human experiment/ or human/) | 2575630 |
|  | 43 or 44 | 2643944 |
|  | 42 not 45 | 5920327 |
|  | 22 and 46 | 1061 |
|  | conference*.pt. or conference abstract/ or preprint.pt. | 5610751 |
|  | 47 not 48 | 738 |
|  | [**PsycINFO 1806 to present**](https://ovidsp.ovid.com/ovidweb.cgi?T=JS&NEWS=N&PAGE=main&SHAREDSEARCHID=4bWEYkY88ImO8zW8kjvg48q8ikiv6SlpcAAmdXap3dawcbGPQzkUEMdyf0MLAoTJo) | **Results** |
| 1 | exp affective disorders/ | 171227 |
| 2 | exp anxiety disorders/ | 42474 |
| 3 | exp anxiety/ or "depression (emotion)"/ | 110407 |
| 4 | exp psychosis/ | 127693 |
| 5 | Mental Disorders/ | 95753 |
| 6 | psychiatric patients/ | 29434 |
| 7 | (depress* or mood? or dysthymi* or anxiet* or anxious or phobia? or psychos?s or psychotic? or schizophren*).ti,ab,hw,id. | 690317 |
| 8 | (((obsessive compulsive or panic or phobic or stress) adj2 disorder?) or (post-traumatic stress or post-traumatic distress or posttraumatic stress or posttraumatic distress or ptsd or ptdd)).ti,ab,hw,id. | 89757 |
| 9 | ((mental* or psycholog* or psychiatric) adj2 (health or ill* or disorder?)).ti,hw,id. | 283199 |
| 10 | 1 or 2 or 3 or 4 or 6 or 7 or 8 or 9 | 952852 |
| 11 | (prebiotic? or pre-biotic? or probiotic? or pro-biotic? or synbiotic? or syn-biotic?).mp. | 624 |
| 12 | (((B or Bacillus) adj coagulans) or B-GOS or bifidobacteria or ((B or Bifidobacterium) adj (animalis or bifidium or bifidus or breve or lactis or longum)) or Bimuno or galactooligosaccharide or ((C or Clostridium) adj butyricum) or ((F or faecalibacterium) adj prausnitzii) or FOS or fructooligosaccharide or GOS or inulin or ((l or lactobacillus) adj (plantarum or acidofilus or acidophilus or brevis or Bulgari* or cas?ei or fermentum or gasseri or helveticus or paraca?sei or pentosus or reuteri or rhamnosus or salivarius or delbrueckii)) or ((l or lactococcus) adj lactis) or ((S or saccharomyces) adj boulardii) or ((S or Streptococ?us) adj (salivarius or thermophiles or thermophilus)) or XOS).mp. | 7164 |
| 13 | (Actinobacteria or Akkermansia or Atopobium or Bacteroidaceae or Bacteroides or Bacteroidetes or Bifidobacteriaceae or Bifidobacterium or Clostridiaceae or Clostridium or Corynebacterium or Dialister or Enterobacteriaceae or Enterococcus or Escherichia or Euryarchaeota or Faecalibacterium or Firmicutes or Fusobacteria or Lachnospiraceae or Lactobacillus or Methanobrevibacter or Oscillospira or Peptostreptococcaceae or Prevotella or Prevotellaceae or Propionibacterium or Proteobacteria or Rikenellaceae or Ruminococcaceae or Ruminococcus or Sneathia or Staphylococcus or Streptococcus or Veillonella or Verrucomicrobia).ti,hw,id. | 637 |
| 14 | gastrointestinal microbiota/ and supplement*.mp. | 40 |
| 15 | (((gut or gastrointestin* or gi or intestinal) adj (microb* or flora)) and supplement*).mp. | 160 |
| 16 | 11 or 12 or 13 or 14 or 15 | 8252 |
| 17 | 10 and 16 | 1676 |
| 18 | random*.ti,ab,hw,id. | 238229 |
| 19 | trial*.ti,ab,hw,id. | 214021 |
| 20 | controlled stud*.ti,ab,hw,id. | 13738 |
| 21 | placebo*.ti,ab,hw,id. | 44201 |
| 22 | ((singl* or doubl* or trebl* or tripl*) and (blind* or mask*)).ti,ab,hw,id. | 32351 |
| 23 | (cross over or crossover or factorial* or latin square).ti,ab,hw,id. | 34920 |
| 24 | (assign* or allocat* or volunteer*).ti,ab,hw,id. | 188361 |
| 25 | treatment effectiveness evaluation/ | 27877 |
| 26 | mental health program evaluation/ | 2339 |
| 27 | exp experimental design/ | 63164 |
| 28 | (clinical trial or treatment outcome).md. | 58267 |
| 29 | 18 or 19 or 20 or 21 or 22 or 23 or 24 or 25 or 26 or 27 or 28 | 608396 |
| 30 | 17 and 29 | 177 |
|  | **CINAHL Query** | **Results** |
| 1 | (MH "Psychotic Disorders+") OR (MH "Mental Disorders") OR (MH "Affective Disorders+") OR (MH "Anxiety Disorders+") OR (MH "Anxiety+") OR (MH "Psychiatric Patients") | 394,526 |
| 2 | ( ((TI depress* OR AB depress* OR SU depress*) OR (TI mood# OR AB mood# OR SU mood#) OR (TI dysthymi* OR AB dysthymi* OR SU dysthymi*) OR (TI anxiet* OR AB anxiet* OR SU anxiet*) OR (TI anxious OR AB anxious OR SU anxious) OR (TI phobia# OR AB phobia# OR SU phobia#) OR (TI psychos#s OR AB psychos#s OR SU psychos#s) OR (TI psychotic# OR AB psychotic# OR SU psychotic#) OR (TI schizophren* OR AB schizophren* OR SU schizophren*)) ) OR ( ((((TI "obsessive compulsive" OR AB "obsessive compulsive" OR SU "obsessive compulsive") OR (TI panic OR AB panic OR SU panic) OR (TI phobic OR AB phobic OR SU phobic) OR (TI stress OR AB stress OR SU stress)) N2 (TI disorder# OR AB disorder# OR SU disorder#)) OR ((TI "post-traumatic stress" OR AB "post-traumatic stress" OR SU "post-traumatic stress") OR (TI "post-traumatic distress" OR AB "post-traumatic distress" OR SU "post-traumatic distress") OR (TI "posttraumatic stress" OR AB "posttraumatic stress" OR SU "posttraumatic stress") OR (TI "posttraumatic distress" OR AB "posttraumatic distress" OR SU "posttraumatic distress") OR (TI ptsd OR AB ptsd OR SU ptsd) OR (TI ptdd OR AB ptdd OR SU ptdd))) ) OR ( (((TI mental*) OR (TI psycholog*) OR (TI psychiatric)) N2 ((TI health) OR (TI ill*) OR (TI disorder#))) ) | 428,638 |
| 3 | 1 OR 2 | 551,939 |
| 4 | (MH "Prebiotics+") OR (MH "Probiotics+") | 10,081 |
| 5 | (MH "Lactobacillus+") OR (MH "Bifidobacterium") OR (MH "Streptococcus Salivarius") OR (MH "Faecalibacterium") | 4,020 |
| 6 | ( ((TI prebiotic# OR AB prebiotic# OR SU prebiotic#) OR (TI pre-biotic# OR AB pre-biotic# OR SU pre-biotic#) OR (TI probiotic# OR AB probiotic# OR SU probiotic#) OR (TI pro-biotic# OR AB pro-biotic# OR SU pro-biotic#) OR (TI synbiotic# OR AB synbiotic# OR SU synbiotic#) OR (TI syn-biotic# OR AB syn-biotic# OR SU syn-biotic#)) ) OR ( ((((TI B OR AB B OR SU B) OR (TI Bacillus OR AB Bacillus OR SU Bacillus)) W1 (TI coagulans OR AB coagulans OR SU coagulans)) OR (TI B-GOS OR AB B-GOS OR SU B-GOS) OR (TI bifidobacteria OR AB bifidobacteria OR SU bifidobacteria) OR (((TI B OR AB B OR SU B) OR (TI Bifidobacterium OR AB Bifidobacterium OR SU Bifidobacterium)) W1 ((TI animalis OR AB animalis OR SU animalis) OR (TI bifidium OR AB bifidium OR SU bifidium) OR (TI bifidus OR AB bifidus OR SU bifidus) OR (TI breve OR AB breve OR SU breve) OR (TI lactis OR AB lactis OR SU lactis) OR (TI longum OR AB longum OR SU longum))) OR (TI Bimuno OR AB Bimuno OR SU Bimuno) OR (TI galactooligosaccharide OR AB galactooligosaccharide OR SU galactooligosaccharide) OR (((TI C OR AB C OR SU C) OR (TI Clostridium OR AB Clostridium OR SU Clostridium)) W1 (TI butyricum OR AB butyricum OR SU butyricum)) OR (((TI F OR AB F OR SU F) OR (TI faecalibacterium OR AB faecalibacterium OR SU faecalibacterium)) W1 (TI prausnitzii OR AB prausnitzii OR SU prausnitzii)) OR (TI FOS OR AB FOS OR SU FOS) OR (TI fructooligosaccharide OR AB fructooligosaccharide OR SU fructooligosaccharide) OR (TI GOS OR AB GOS OR SU GOS) OR (TI inulin OR AB inulin OR SU inulin) OR (((TI l OR AB l OR SU l) OR (TI lactobacillus OR AB lactobacillus OR SU lactobacillus)) W1 ((TI plantarum OR AB plantarum OR SU plantarum) OR (TI acidofilus OR AB acidofilus OR SU acidofilus) OR (TI acidophilus OR AB acidophilus OR SU acidophilus) OR (TI brevis OR AB brevis OR SU brevis) OR (TI Bulgari* OR AB Bulgari* OR SU Bulgari*) OR (TI cas#ei OR AB cas#ei OR SU cas#ei) OR (TI fermentum OR AB fermentum OR SU fermentum) OR (TI gasseri OR AB gasseri OR SU gasseri) OR (TI helveticus OR AB helveticus OR SU helveticus) OR (TI paraca#sei OR AB paraca#sei OR SU paraca#sei) OR (TI pentosus OR AB pentosus OR SU pentosus) OR (TI reuteri OR AB reuteri OR SU reuteri) OR (TI rhamnosus OR AB rhamnosus OR SU rhamnosus) OR (TI salivarius OR AB salivarius OR SU salivarius) OR (TI delbrueckii OR AB delbrueckii OR SU delbrueckii))) OR (((TI l OR AB l OR SU l) OR (TI lactococcus OR AB lactococcus OR SU lactococcus)) W1 (TI lactis OR AB lactis OR SU lactis)) OR (((TI S OR AB S OR SU S) OR (TI saccharomyces OR AB saccharomyces OR SU saccharomyces)) W1 (TI boulardii OR AB boulardii OR SU boulardii)) OR (((TI S OR AB S OR SU S) OR (TI Streptococ#us OR AB Streptococ#us OR SU Streptococ#us)) W1 ((TI salivarius OR AB salivarius OR SU salivarius) OR (TI thermophiles OR AB thermophiles OR SU thermophiles) OR (TI thermophilus OR AB thermophilus OR SU thermophilus))) OR (TI XOS OR AB XOS OR SU XOS)) ) OR ( ((TI Actinobacteria) OR (TI Akkermansia) OR (TI Atopobium) OR (TI Bacteroidaceae) OR (TI Bacteroides) OR (TI Bacteroidetes) OR (TI Bifidobacteriaceae) OR (TI Bifidobacterium) OR (TI Clostridiaceae) OR (TI Clostridium) OR (TI Corynebacterium) OR (TI Dialister) OR (TI Enterobacteriaceae) OR (TI Enterococcus) OR (TI Escherichia) OR (TI Euryarchaeota) OR (TI Faecalibacterium) OR (TI Firmicutes) OR (TI Fusobacteria) OR (TI Lachnospiraceae) OR (TI Lactobacillus) OR (TI Methanobrevibacter) OR (TI Oscillospira) OR (TI Peptostreptococcaceae) OR (TI Prevotella) OR (TI Prevotellaceae) OR (TI Propionibacterium) OR (TI Proteobacteria) OR (TI Rikenellaceae) OR (TI Ruminococcaceae) OR (TI Ruminococcus) OR (TI Sneathia) OR (TI Staphylococcus) OR (TI Streptococcus) OR (TI Veillonella) OR (TI Verrucomicrobia)) ) | 34,468 |
| 7 | (MH "Gut Microbiota") AND (TI supplement* OR AB supplement* OR SU supplement*) | 970 |
| 8 | ((((TI gut OR AB gut OR SU gut) OR (TI gastrointestin* OR AB gastrointestin* OR SU gastrointestin*) OR (TI gi OR AB gi OR SU gi) OR (TI intestinal OR AB intestinal OR SU intestinal)) W1 ((TI microb* OR AB microb* OR SU microb*) OR (TI flora OR AB flora OR SU flora))) AND (TI supplement* OR AB supplement* OR SU supplement*)) | 1,929 |
| 9 | 4 OR 5 OR 6 OR 7 OR 8 | 36,154 |
| 10 | 3 AND 9 | 956 |
| 11 | MH randomized controlled trials OR MH double-blind studies OR MH single-blind studies OR MH random assignment OR MH pretest-posttest design OR MH cluster sample OR TI (randomised OR randomized) OR AB (random*) OR TI (trial) OR (MH (sample size) AND AB (assigned OR allocated OR control)) OR MH (placebos) OR PT (randomized controlled trial) OR AB (control W5 group) OR MH (crossover design) OR MH (comparative studies) OR AB (cluster W3 RCT) | 1,002,601 |
| 12 | (MH animals+ OR MH (animal studies) OR TI (animal model*)) NOT MH (human) | 211,962 |
| 13 | 11 NOT 12 | 955,476 |
| 14 | 10 AND 13 | 198 |
|  | **Cochrane** | **Results** |
| 1 | MeSH descriptor: [Depressive Disorder] explode all trees | 15055 |
| 2 | MeSH descriptor: [Anxiety Disorders] explode all trees | 8908 |
| 3 | MeSH descriptor: [Depression] explode all trees | 18299 |
| 4 | MeSH descriptor: [Anxiety] explode all trees | 13016 |
| 5 | MeSH descriptor: [Schizophrenia Spectrum and Other Psychotic Disorders] explode all trees | 12071 |
| 6 | MeSH descriptor: [Mental Disorders] this term only | 4913 |
| 7 | (depress*:ti,ab,kw OR mood?:ti,ab,kw OR dysthymi*:ti,ab,kw OR anxiet*:ti,ab,kw OR anxious:ti,ab,kw OR phobia?:ti,ab,kw OR psychos?s:ti,ab,kw OR psychotic?:ti,ab,kw OR schizophren*:ti,ab,kw) | 170340 |
| 8 | ((("obsessive compulsive":ti,ab,kw OR panic:ti,ab,kw OR phobic:ti,ab,kw OR stress:ti,ab,kw) NEAR/2 disorder?:ti,ab,kw) OR ("post-traumatic stress":ti,ab,kw OR "post-traumatic distress":ti,ab,kw OR "posttraumatic stress":ti,ab,kw OR "posttraumatic distress":ti,ab,kw OR ptsd:ti,ab,kw OR ptdd:ti,ab,kw)) | 15002 |
| 9 | ((mental*:ti OR psycholog*:ti OR psychiatric:ti) NEAR/2 (health:ti OR ill*:ti OR disorder?:ti)) | 7270 |
| 10 | #1 OR #2 OR #3 OR #4 OR #5 OR #6 OR #7 OR #8 OR #9 | 182234 |
| 11 | MeSH descriptor: [Prebiotics] explode all trees | 608 |
| 12 | MeSH descriptor: [Probiotics] explode all trees | 3027 |
| 13 | MeSH descriptor: [Lactobacillus] explode all trees | 1543 |
| 14 | MeSH descriptor: [Lactococcus] explode all trees | 35 |
| 15 | MeSH descriptor: [Streptococcus salivarius] explode all trees | 25 |
| 16 | MeSH descriptor: [Streptococcus thermophilus] explode all trees | 87 |
| 17 | MeSH descriptor: [Bifidobacterium] explode all trees | 1117 |
| 18 | MeSH descriptor: [Bacillus coagulans] explode all trees | 31 |
| 19 | MeSH descriptor: [Clostridium butyricum] explode all trees | 35 |
| 20 | MeSH descriptor: [Faecalibacterium prausnitzii] explode all trees | 9 |
| 21 | (((B:ti,ab,kw OR Bacillus:ti,ab,kw) NEXT coagulans:ti,ab,kw) OR B-GOS:ti,ab,kw OR bifidobacteria:ti,ab,kw OR ((B:ti,ab,kw OR Bifidobacterium:ti,ab,kw) NEXT (animalis:ti,ab,kw OR bifidium:ti,ab,kw OR bifidus:ti,ab,kw OR breve:ti,ab,kw OR lactis:ti,ab,kw OR longum:ti,ab,kw)) OR Bimuno:ti,ab,kw OR galactooligosaccharide:ti,ab,kw OR ((C:ti,ab,kw OR Clostridium:ti,ab,kw) NEXT butyricum:ti,ab,kw) OR ((F:ti,ab,kw OR faecalibacterium:ti,ab,kw) NEXT prausnitzii:ti,ab,kw) OR FOS:ti,ab,kw OR fructooligosaccharide:ti,ab,kw OR GOS:ti,ab,kw OR inulin:ti,ab,kw OR ((l:ti,ab,kw OR lactobacillus:ti,ab,kw) NEXT (plantarum:ti,ab,kw OR acidofilus:ti,ab,kw OR acidophilus:ti,ab,kw OR brevis:ti,ab,kw OR Bulgari*:ti,ab,kw OR cas?ei:ti,ab,kw OR fermentum:ti,ab,kw OR gasseri:ti,ab,kw OR helveticus:ti,ab,kw OR paraca?sei:ti,ab,kw OR pentosus:ti,ab,kw OR reuteri:ti,ab,kw OR rhamnosus:ti,ab,kw OR salivarius:ti,ab,kw OR delbrueckii:ti,ab,kw)) OR ((l:ti,ab,kw OR lactococcus:ti,ab,kw) NEXT lactis:ti,ab,kw) OR ((S:ti,ab,kw OR saccharomyces:ti,ab,kw) NEXT boulardii:ti,ab,kw) OR ((S:ti,ab,kw OR Streptococ?us:ti,ab,kw) NEXT (salivarius:ti,ab,kw OR thermophiles:ti,ab,kw OR thermophilus:ti,ab,kw)) OR XOS:ti,ab,kw) | 8778 |
| 22 | (Actinobacteria:ti OR Akkermansia:ti OR Atopobium:ti OR Bacteroidaceae:ti OR Bacteroides:ti OR Bacteroidetes:ti OR Bifidobacteriaceae:ti OR Bifidobacterium:ti OR Clostridiaceae:ti OR Clostridium:ti OR Corynebacterium:ti OR Dialister:ti OR Enterobacteriaceae:ti OR Enterococcus:ti OR Escherichia:ti OR Euryarchaeota:ti OR Faecalibacterium:ti OR Firmicutes:ti OR Fusobacteria:ti OR Lachnospiraceae:ti OR Lactobacillus:ti OR Methanobrevibacter:ti OR Oscillospira:ti OR Peptostreptococcaceae:ti OR Prevotella:ti OR Prevotellaceae:ti OR Propionibacterium:ti OR Proteobacteria:ti OR Rikenellaceae:ti OR Ruminococcaceae:ti OR Ruminococcus:ti OR Sneathia:ti OR Staphylococcus:ti OR Streptococcus:ti OR Veillonella:ti OR Verrucomicrobia:ti) | 5733 |
| 23 | MeSH descriptor: [Gastrointestinal Microbiome] explode all trees | 1307 |
| 24 | (((gut:ti,ab,kw OR gastrointestin*:ti,ab,kw OR gi:ti,ab,kw OR intestinal:ti,ab,kw) NEXT (microb*:ti,ab,kw OR flora:ti,ab,kw)) AND supplement*:ti,ab,kw) | 1949 |
| 25 | #11 OR #12 OR #13 OR #14 OR #15 OR #16 OR #17 OR #18 OR #19 OR #20 OR #21 OR #22 OR #23 OR #24 | 15143 |
| 26 | #10 AND #25 | 671 |
| 27 | #10 and #25 in Cochrane Reviews | 6 |
| 28 | #10 and #25 in Trials | 665 |
